# Effects of low wildfire burn severity due to pre-fire shrub thinning on the chaparral soil bacteriome in the Santa Monica Mountains of Southern California

**DOI:** 10.1128/spectrum.00185-25

**Published:** 2025-06-30

**Authors:** Mariah Macias, Mari R. Irving, Katelyn M. Bandow, Kaitlyn Kim, Cecilia Heredia, Courtney A. Hoskinson, Nina R. Duchild, Michael T. Nicholas, Lindsey M. Marian, Camille K. Sicangco, Karagan L. Smith, Stephen D. Davis, Helen I. Holmlund, Leah T. Stiemsma

**Affiliations:** 1 Natural Science Division, Pepperdine University 166578, Malibu, California, USA; 2 Department of Ecology, Evolution, and Marine Biology, University of California Santa Barbara 166653, Santa Barbara, California, USA; 3 Department of Microbiology & Immunology, University of British Columbia 198130, Vancouver, British Columbia, Canada; 4 Hawkesbury Institute for the Environment, Western Sydney University 283861, Richmond, New South Wales, Australia; 5 Department of Ecology, Behaviour, and Evolution, University of California San Diego 315225, La Jolla, California, USA; Instituto de Ecología, A.C. (INECOL), Pátzcuaro, Michoacán, Mexico

**Keywords:** soil microbiome, soil bacteriome, low and high severity burn, chaparral microbiome, wildfire severity

## Abstract

Our objective was to study the longitudinal effect of decreased burn severity due to vegetation-type conversion (VTC) induced by chaparral shrub thinning prior to the Woolsey wildfire (November 2018) on soil chemistry and bacteriome composition and function. We compared soils from two study sites on the Malibu campus of Pepperdine University in the Santa Monica Mountains: one site had dense, unaltered chaparral shrubland and experienced a 4.5-fold increase in vegetation burn severity (high severity burn) compared to an adjacent altered site where the vegetative fuel load was 80% less prior to the fire (low severity burn). We analyzed soil nutrient concentrations and pH in 2019 and 2021 and soil respiration, measured by CO_2_ efflux, in 2019, 2020, and 2021. DNA was isolated from soil samples collected in 2019, 2020, 2021, and 2023 for Illumina Miseq paired-end 16S V3-V4 sequencing. We predicted the functional bacteriome from the 16S data using PICRUSt2. Relative to high severity soils, low severity soils showed decreased nutrient concentrations, pH, and % organic matter in 2019. The low severity burned site showed greater compositional stability over time, with increased pyrophilous taxa in 2021 and 2023 (*Massilia*, *Conexibacter*, etc.). High severity burned soils showed decreased metabolic capacity over time. We identified correlations between bacterial taxa and diversity and functional pathways, which remained only in the high severity soil samples after stratification. Our findings contribute to an improved understanding of bacterial succession in soil from sites that experienced VTC prior to wildfire, highlighting microbial ecological implications for fire management strategies.

**IMPORTANCE:**

Along with increased fire frequency, the wildfire-urban interface has been expanding, requiring the need for fire mitigation strategies, such as pre-fire vegetation thinning near urban structures. Pre-fire vegetation thinning contributes to vegetation-type conversion and decreases burn severity, but its effect on the soil microenvironment is largely unknown. Here, we compared soil sites that experienced burns of different severity due to pre-fire vegetation thinning and vegetation-type conversion at one site but not the other. We identified changes in soil chemistry and longitudinal shifts in soil bacterial abundance and metabolic capacity that are associated with decreased burn severity due to pre-fire vegetation-type conversion. Our work contributes to improved understanding of the effects of pre-fire vegetation thinning to manage wildfire impact on urban structures on the soil microenvironment. These findings demonstrate ecological implications for fire management strategies and recovery of the chaparral ecosystems following wildfire.

## INTRODUCTION

Coastal Southern California represents one of five Mediterranean-type ecosystems (MTE) in the world (1). MTEs are characterized by hot, dry summers and cool, wet winters, with Santa Ana Foehn-type winds that augment wildfires in fall months (1). These ecosystems historically experienced wildfire every 30–100 years, with plant-life primed for regrowth following wildfire (1). However, the natural landscape of Pepperdine University in Malibu, California, has experienced increased fire frequency since 1923, with some fire return intervals as low as only three years between fires (2, 3). The Woolsey Fire in November 2018 was the largest wildfire in the region in recorded history, burning nearly one-half of the entire range of the Santa Monica Mountains (SMM) (4).

Increased fire frequency in the SMM due to anthropogenic climate change has resulted in vegetation type-conversion (permanent or semi-permanent replacement of native shrubs with invasive grasses, VTC) (2, 5, 6). With increased fire frequency, obligate seeding species, including evergreen sclerophyllous chaparral shrubs, are unable to mature, produce more seeds, and restore soil seed banks prior to subsequent fires (2, 3, 7–9). Thus, the region is becoming primarily populated by invasive forbs and grasses (1, 2). Invasive forbs and grasses contain less combustible fuel than woody shrubs and, thus, burn at lower temperatures, killing fewer non-refractory seeds of exotics while failing to heat stimulate the germination of refractory seeds of native chaparral species (10). Thus, VTC in the SMMs is both the result of and a contributor to the decreased fire return intervals observed in the SMMs (1, 2).

Alongside this increased frequency of wildfire in the SMMs, the wildland-urban interface (WUI) has been expanding due to increased urban sprawl in recent decades in the USA (11) and globally (12, 13). Typical fire management strategies to protect nearby urban structures involve pre-fire vegetation thinning, which reduces the combustible fuel load and reduces fire severity (14). However, as Keeley predicted in 1999 (15), urban sprawl and these associated fire management strategies are having profound ecological impacts on the natural landscape (16). The Davis lab has recorded evidence of VTC in the natural landscape of Pepperdine University since the 1990s (1, 2). However, the effect of VTC due to pre-fire mitigation strategies on the soil microbiome has yet to be addressed.

Wildfire also shapes the ecology and functionality of the soil microbiome (17, 18). The soil microbiome is key in maintaining plant-life diversity and protection from pathogenic organisms (17, 18). Specifically, soil microbes breakdown of soil organic matter, releasing key soil nutrients (utilized by plants), contributes to nitrogen fixation, and heavily influences soil carbon cycling and regeneration, all of which contribute to plant growth (17–19). Recent findings report the role of the soil microbiome in degrading pyrogenic organic matter and increased nitrogen cycling following wildfire, highlighting the role of specific bacterial taxa in plant life’s response to wildfire (20). Studies report bacterial dysbiosis in response to wildfires characterized by the loss of heat-sensitive taxa and an increased abundance of pyrophilous taxa, which are often heat-resistant, fast-growing, and/or capable of sporulating (17, 18, 21, 22). Wildfires also alter the chemistry of soil, favoring growth of specific taxa, which can further alter the soil microenvironment and lead to long-lasting soil dysbiosis and altered microbial succession (17, 23). Since soil microbes directly interact with surrounding plant-life, there is concern that increased fire frequency will alter the soil microenvironment such that post-fire recovery of the ecosystem is threatened (21).

Current research reports altered microbial succession post-wildfire in the chaparral environment (22, 23). In the SMM specifically, Cox et al. report altered soil microbial composition across habitats populated by invasive or native plant species (24). This group analyzed the soil microbiome before and after the Woolsey fire in 2018 and found that irrespective of wildfire, these habitat differences persist (24). Research also shows differential effects on the soil microenvironment, specifically altered functional diversity, due to fire intensity (cooler vs hotter burns, which result in altered wildfire severity levels) (25, 26). However, microbial succession as it relates to pre-shrub thinning induced VTC and ultimately reduced fire severity in chaparral shrub communities is largely unknown.

The objective of this study was to assess the longitudinal effect of lower wildfire severity due to VTC induced by chaparral shrub thinning prior to wildfire on the soil microenvironment and bacteriome after the Woolsey wildfire of November 2018. We compared soil from two study sites within the natural landscape of the Pepperdine University campus in Malibu, California. Though both sites burned during the Woolsey fire, one site’s vegetative fuel load was 80% thinned by fire-management personnel prior to the burn as part of a mitigation strategy to protect nearby structures, often described as wildfire defensible space. This site was primarily populated by invasive forbs and grasses, having experienced VTC prior to the Woolsey fire. The other site was not thinned and represented a natural landscape dominated by densely spaced, nearly impenetrable stands of evergreen chaparral shrubs of coastal exposures of the SMMs. Due to pre-fire shrub thinning 60 m distant from the nearest structures in the WUI, the severity of the Woosley fire at each site differed markedly, with the high severity burn site experiencing nearly a 4.5-fold increase in vegetation burn severity relative to the low severity burn site that was pre-thinned. Burn severity can be described as the amount of above or belowground biomass that is consumed during a fire and varies depending on the fire’s temperature and rate at which it moves across the landscape (27). The chaparral vegetation and soil microbiome are highly intertwined (17, 18), and thus, we hypothesized that lower vegetation burn severity due to pre-fire vegetation thinning and VTC would result in longitudinal alterations in soil chemistry, bacterial recovery, and bacterial succession. Our findings suggest that decreased burn severity is associated with decreased soil nutrient availability and pH. The low severity burned site showed greater compositional stability over time. Furthermore, the low and high severity burned sites showed similarities in global bacterial diversity in 2019 and 2020. However, in the later years following the wildfire (2021 and 2023), the sites diverged. In 2021 and 2023, high severity burned sites displayed more diverse taxa accompanied by decreased metabolic capacity. Taken together, not only does VTC prior to fire result in diminished fire severity, but it also contributes to longitudinal changes to the soil microenvironment and bacteriome, which represent profound implications for fire management strategies in the context of rapidly increasing WUI (11).

## RESULTS

### Lower vegetation burn severity due to pre-fire VTC does not alter soil respiration

**Fig 1:**
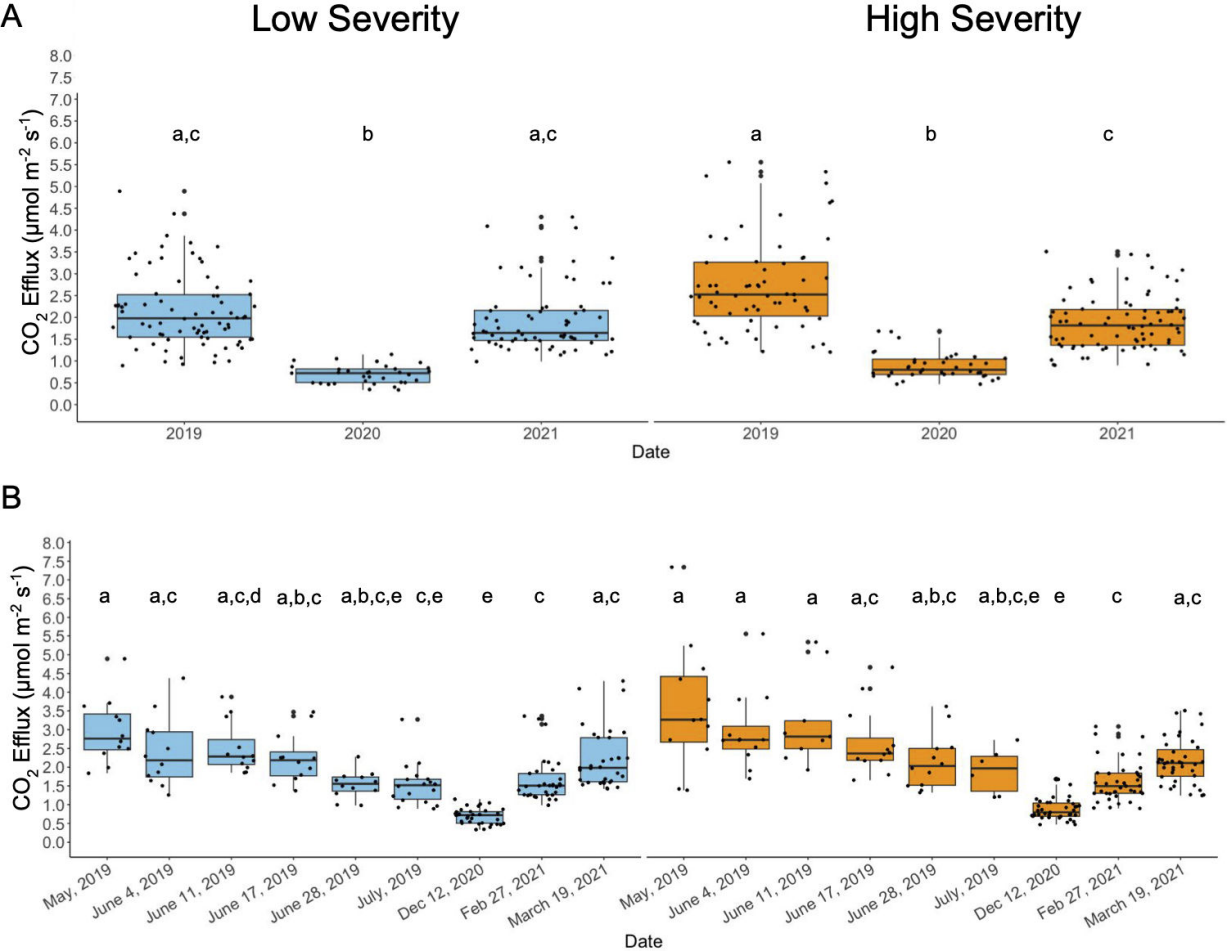
Decreased burn severity due to vegetation thinning decreases soil respiration. (**A**) Soil respiration measurements for 2019, 2020, and 2021 across low (blue) and high (orange) severity burn sites. (**B**) Respiration measurements throughout 2019, 2020, and 2021 by specific date collected. Repeated letters represent no statistical significance between groups at an adjusted *P* < 0.05 (Kruskal-Wallis with post-hoc Dunn’s, Bonferroni adjustment). 2019 high *n* = 62 (May *n* = 11, June 4 *n* = 12, June 11 *n* = 9, June 17 *n* = 12, June 28 *n* = 12, July *n* = 6), low *n* = 72 (*n* = 12 for all time points), 2020 high *n* = 39, 2020 low *n* = 33, 2021 high *n* = 78 (February *n* = 39, March *n* = 39), 2021 low *n* = 66 (February *n* = 22, March *n* = 33).

Soil respiration was measured in terms of CO_2_ efflux from the soil surface from May 2019 to March 2021. Regardless of wildfire burn severity, measurements taken on 12 December 2020, displayed the lowest respiration rates relative to collections taken in both 2019 and 2021 (Fig. 1), likely due to little rainfall (reported 2.5 mm of rainfall [28]) during Fall 2020. Soil respiration rates increased in both low and high severity burn sites from 2020 to 2021 (Fig. 1A). When comparing paired time points (i.e., May 2019 for low severity compared with May 2019 for high severity), even though there is increased respiration in the high severity burn site relative to the low severity burn site, this difference was not statistically significant at an adjusted *P* value < 0.05 (Bonferonni adjustment, Fig. 1B). In conclusion, vegetation thinning prior to wildfire, which resulted in altered burn severity levels across the sites, does not appear to alter soil respiration rates.

### Decreased burn severity due to pre-fire VTC alters soil nutrient content and pH

**Fig 2:**
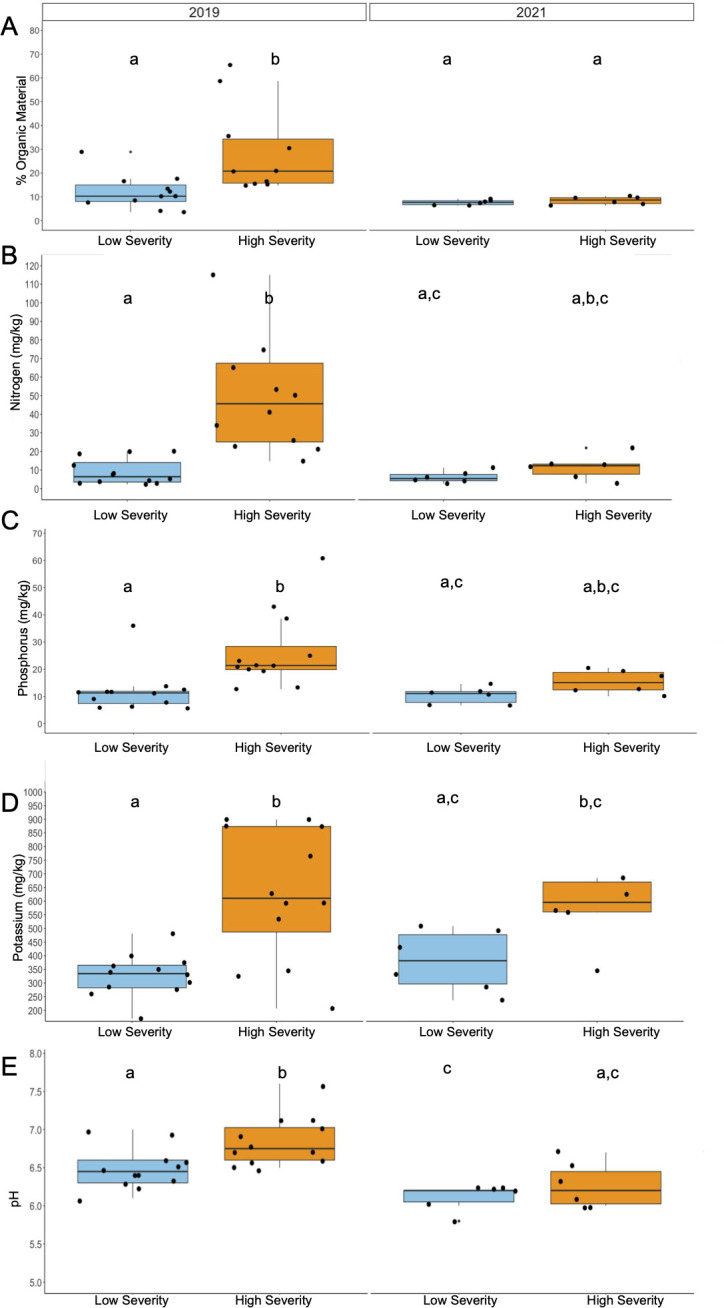
Decreased burn severity due to vegetation thinning alters soil nutrient content and pH. (**A**) Percent organics, (**B**) nitrogen concentration, (**C**) phosphorus concentration, (**D**) potassium concentration, and (**E**) pH in low (blue) and high (orange) severity burned soils in 2019 and 2021. Repeated letters represent no statistical significance between groups at an adjusted *P* < 0.05 (Kruskal-Wallis with post-hoc Dunn’s, Bonferroni adjustment for % organics, N, K, and P; ANOVA and post-hoc Tukey for pH). 2019 high *n* = 10, 2019 low *n* = 11. 2021 high *n* = 12, 2021 low *n* = 12.

We measured percent organic matter, concentrations of key mineral elements for bacteria and vascular plants (nitrogen [N], phosphorus [P], and potassium [K]), and soil pH in high severity and low severity burned sites in 2019 and 2021. Percent organic material was decreased in low severity burned soil in 2019 (*P* = 0.049, File S1; Fig. 2A). Low severity burned soils also showed decreased nutrient availability, such that N, P, and K were all decreased in low severity burned soils in 2019 (N *P* = 0.0002, P *P* = 0.0004, K *P* = 0.01, File S1; Fig. 2B through D).

Additionally, low severity burned soils maintained similar concentrations of N, P, and K and percent organics while high severity burned soils showed decreased percent organics (*P* = 0.007) in 2021 relative to 2019 (File S1; Fig. 2A through D). These data are consistent with our respiration measurements, indicating decreased metabolic rates in these soils in the years following the Woolsey wildfire.

These soil nutrients can shift in abundance following wildfire as a result of shifts in soil pH (29). In our study, low severity burned soils showed decreased pH in 2019 relative to high severity burned soils (*P* = 0.017, Fig. 2E; File S1). By 2021, the high and low severity burned soils were similar (File S1; Fig. 2E). Consistent with previous studies (29), soil is more alkaline immediately following wildfire and becomes slightly more acidic over time; the pH of both high and low severity burned soils decreased from 2019 to 2021 (*P* < 0.01, File S1; Fig. 2E). Shifts in soil pH can influence the composition of the soil bacteriome due to increases in bioactivity of soil nutrients (29). Conversely, specific bacterial taxa, as well as overall changes in bacterial diversity, may influence nutrient availability in the soil microenvironment.

### Lower vegetation burn severity resulting from pre-fire VTC was associated with global shifts in soil bacterial diversity

**Fig 3:**
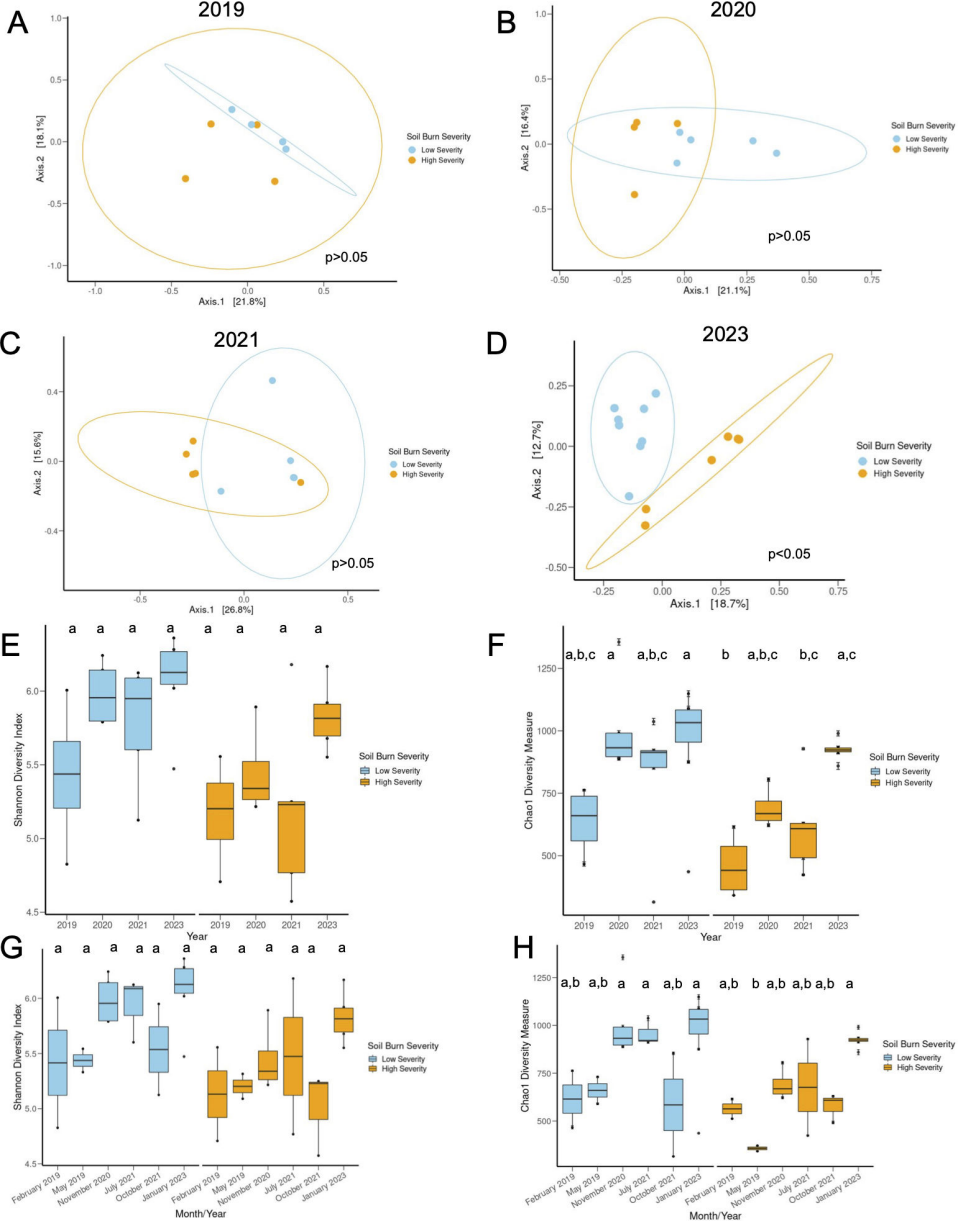
Vegetation burn severity resulting from site-specific pre-fire vegetation thinning is associated with global shifts in soil bacterial diversity. Beta diversity based on unweighted UniFrac distances between hot and cool burned sites in (**A**) 2019 (high vs low PCoA not significant [ns]), (**B**) 2020 (high vs low PCoA ns), (**C**) 2021 (high vs low PCoA ns), and (**D**) 2023 (high vs low PCoA, Permanova *P* < 0.05). Ovals represent confidence intervals. (**E**) Shifts in Shannon diversity and (**F**) Chao1 diversity in high severity (orange) and low severity (blue) burned sites in 2019, 2020, 2021, and 2023. (**G**) Shannon diversity by month and year of collection and (**H**) Chao1 diversity by month and year of collection (Kruskal-Wallis with post-hoc Dunn’s, Bonferroni adjustment for Shannon, ANOVA and post-hoc Tukey for Chao1). February 2019 (*n* = 4, 2 low and 2 high), May 2019 (*n* = 4, 2 low and 2 high), November 2020 (*n* = 9, 5 low and 4 high), July 2021 (*n* = 5, 3 low and 2 high), October 2021 (*n* = 5, 2 low and 3 high), and January 2023 (*n* = 14, 8 low and 6 high).

**Fig 4:**
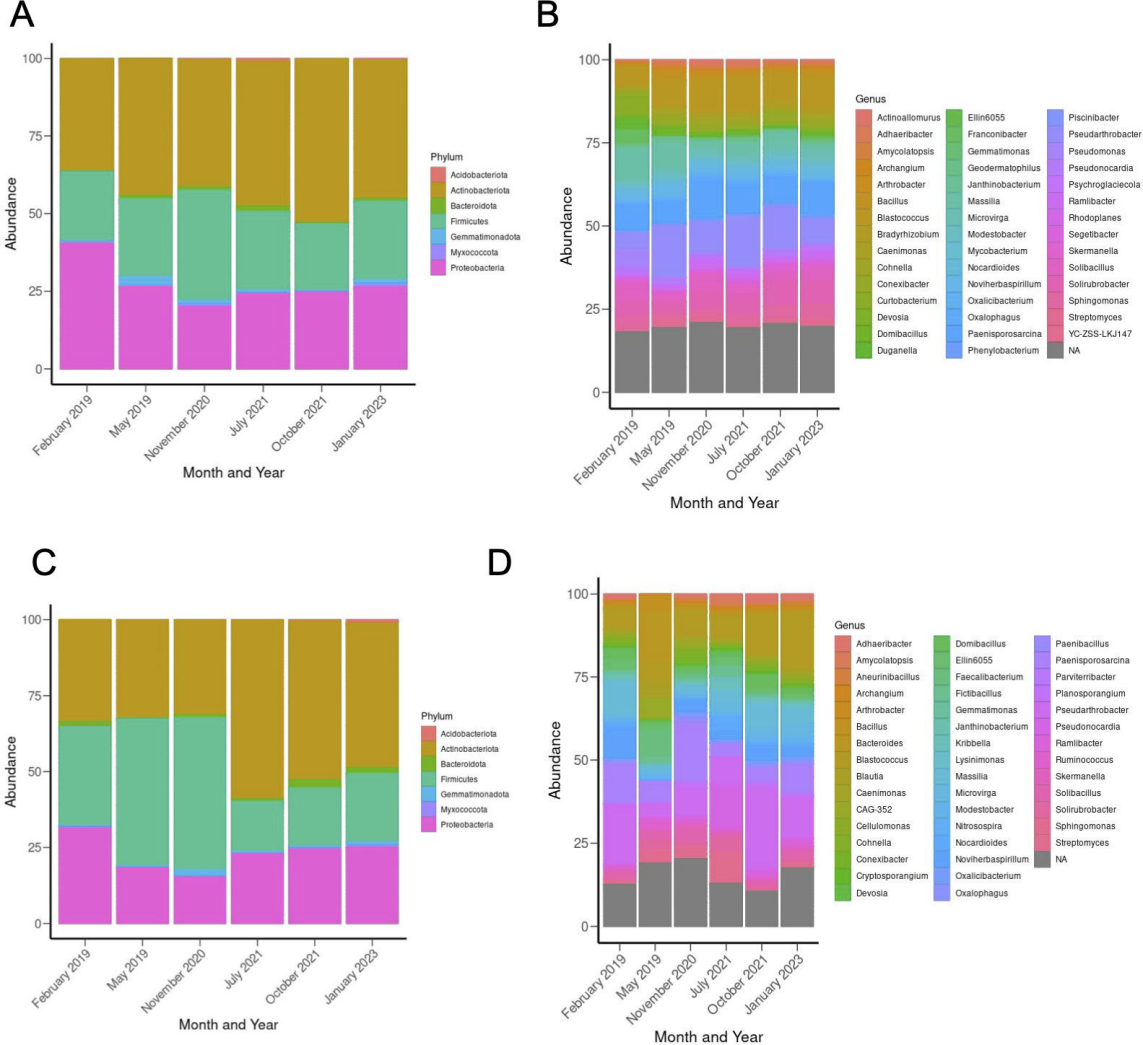
Phylum and genus level shifts in bacterial abundance in low and high severity burn sites. Top 100 ASVs (**A**) phylum level and (**B**) genus level abundances in February and May 2019, November 2020, July and October 2021, and January 2023 in low severity burned soils. Top 100 ASV (**C**) phylum level and (**D**) genus level abundance in February and May 2019, November 2020, July and October 2021, and January 2023 in high severity burned soils. Colors on the relative abundance graphs represent specific bacterial phyla (see legends). The size of the rectangles in the stacked bar plots indicates the percentage of the taxon in the soil during that time point.

Principal coordinate analysis (PCoA) based on unweighted UniFrac (species presence/absence, Fig. 3A through D) and weighted UniFrac (species presence/absence and abundance, File S2) distances highlights increased dissimilarity of high and low severity burn sites in January 2023, approximately 4.25 years following the Woolsey Fire (November, 2018). Specifically, the bacteriome was more similar in 2019 between high and low severity burn sites (*P* > 0.05 for both unweighted and weighted UniFrac, Fig. 4A through D; Files S1 and S2) and more dissimilar in 2023 across high and low severity burn sites (*P* = 0.001 for unweighted and *P* = 0.015 for weighted, Fig. 4D; Files S1 and S2). We also noted increased dissimilarity in the low and high severity sites over time (*P* low severity across years = 0.041 for unweighted and 0.027 for weighted, *P* high severity across years = 0.002 for unweighted, and *P* > 0.05 for weighted, Fig. S3; Files S1 and S2). Figure S3 highlights the increased dissimilarity over time and the influence of burn severity on beta diversity of the soil microbiome.

We report lower Chao1 alpha diversity (bacterial richness) in high severity burn sites in 2019 (Fig. 3B; File S1, *P* = 0.0092), specifically May 2019 (Fig. 3H; File S1, *P* = 0.019) relative to January 2023. We did not identify any differences in alpha diversity within the low severity burn sites across months or years (Fig. 3E through H; File S1). There were also no statistical differences in Shannon diversity across high and low severity burned soils over time (richness and evenness, Fig. 3E and G; File S1). In May 2019, high severity burned soils displayed the lowest bacterial richness of all the soils studied (Fig. 3E through H; File S1) and were significantly different from low severity soils in November 2020 (*P* = 0.005) and July 2021 (*P* = 0.031, Fig. 3H; File S1). There were additional statistical differences in Chao1 diversity between high and low severity burned soils at different collection time points (Fig. 3F and H; File S1). However within the same time point (e.g., 2019 low vs 2019 high), soil burn severity due to pre-fire VTC was not associated with soil bacterial alpha diversity.

### Low wildfire severity due to pre-fire VTC results in more consistent bacterial profiles longitudinally

As an additional measure of bacterial diversity, we conducted an analysis of bacterial relative abundance in the two sites each year (Fig. 4). At the phylum and genus level, the low severity sites displayed more consistent bacterial profiles over time, while the high severity sites underwent a multitude of changes. For the high severity burned sites specifically, Bacteroidota and Acidobacteriota were more abundant in July and October 2021 and January 2023 as compared with the low severity sites (Fig. 4A and C). High severity burned soil presented a large abundance of Firmicutes from February 2019 to November 2020; thereafter, this abundance drops precipitously (Fig. 4C). Low severity sites displayed increased levels of Bacteroidota from May 2019 to July 2021, as well as a large presence of Acidobacteriota in July 2021 (Fig. 4A). A shared characteristic between high and low severity sites is the consistent abundance of Proteobacteria. Proteobacteria were higher in low severity soils, but both high and low severity burned soils showed the highest abundance of this phylum in February 2019 (Fig. 4A and C).

**Fig 5:**
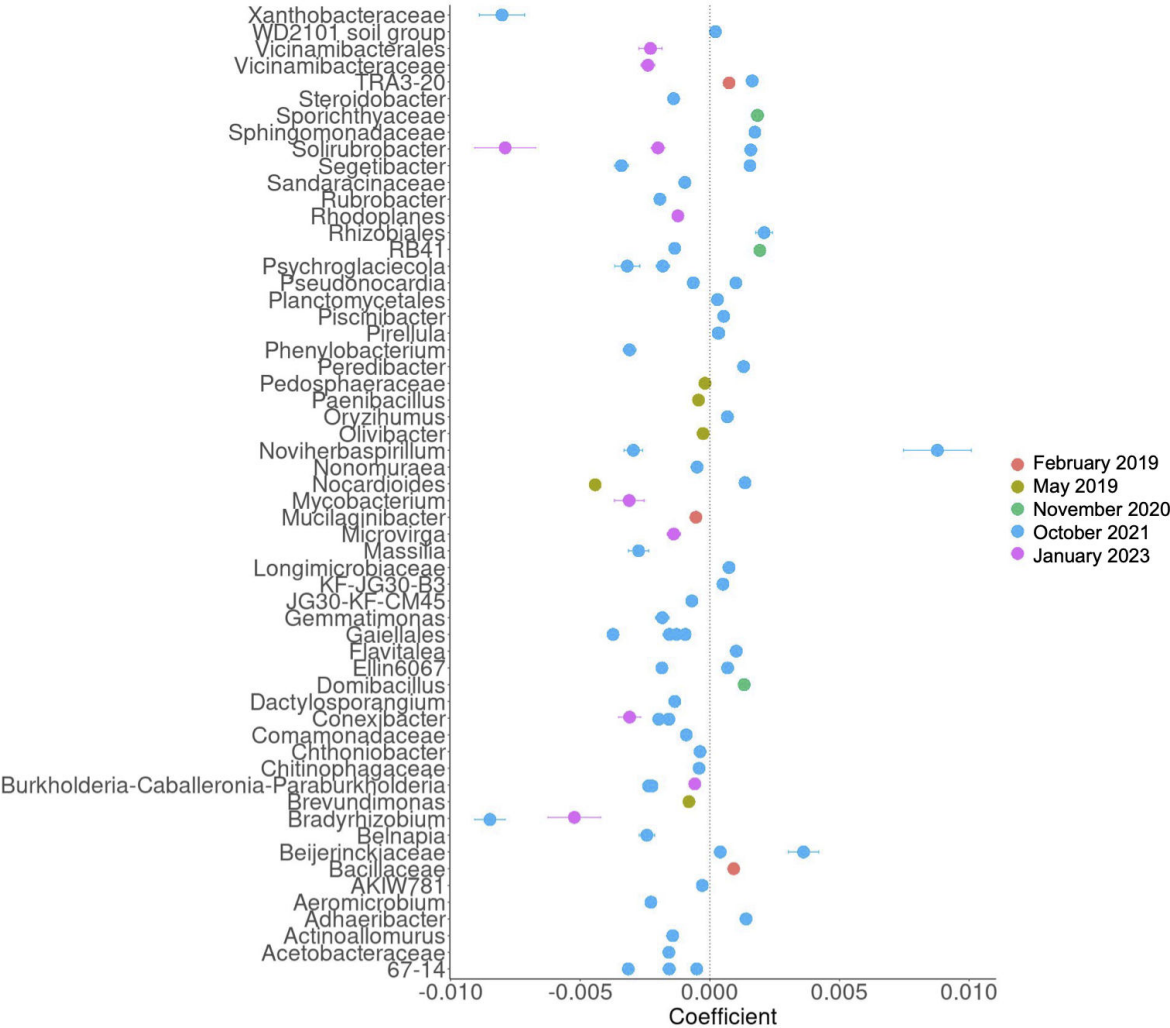
Differentially abundant ASVs in high severity burned soils relative to low severity burned soils. Coefficient is equivalent to the effect size and is a measure of the difference in abundance of the taxa in the high severity burned soil relative to the low severity burned soil. Only ASVs differentially abundant at a *q* value threshold < 0.25 are shown (Benjamin-Hochberg adjustment). ASVs are specified by their lowest taxonomic rank from February 2019 (*n* = 4, 2 low and 2 high), May 2019 (*n* = 4, 2 low and 2 high), November 2020 (*n* = 9, 5 low and 4 high), October 2021 (*n* = 5, 2 low and 3 high), and January 2023 (*n* = 14, 8 low and 6 high).

At the genus level, the bacterial profile consistency in the low severity burn sites over time is more pronounced, as compared with the more dynamic shifts in bacterial abundances over time in the high severity soils (Fig. 4B and D). May 2019 high severity soils showed increased *Bacillus* spp. and a lack of *Adhaeribacter* as compared with low severity burned soils and high severity burn soils in all other time points (Fig. 4B and D). Figure 4A through D displayed aggregated ASV percentages in low and high severity soils across time points. We used MaAsLin2 to run a statistical analysis on single differentially abundant (DA) ASVs, classified to the lowest taxonomic rank possible, between high and low severity burn sites (Fig. 5; File S3) and in the high and low severity sites over time (File S3, *q* value threshold = 0.25). When comparing ASVs within soil sites over time, the high severity burned soils displayed 2,022 DA ASVs and low severity burned soils displayed 1,209 DA ASVs in May 2019, November 2020, July 2021, October 2021, and January 2023 relative to February 2019. The genus *Solirubrobacter* was among the most significantly DA taxa in high severity soils over time, ASVs for which were identified in May 2019, November 2020, and October 2021 (File S3). ASVs for *Edaphobaculum* and *Mucilaginibacter* were identified as DA across all time points in low severity burn soils (File S3).

Within each time point and comparing high to low severity burn soils, Xanthobacteraceae (family, October 2021), *Solirubrobacter* (genus, January 2023), and *Bradyrhizobium* (genus, October 2021 and January 2023) were the lowest in abundance in high severity soils (Fig. 5). *Noviherbaspirillum* (genus, October 2021) was the highest taxon in abundance in high severity soils relative to low severity soils (Fig. 5). In February 2019, Bacillaceae (family) and TRA3-20 (uncultured bacterium) were increased in high severity burned soils, while *Mucilaginibacter* (genus) were decreased in high severity soils relative to low severity soils (Fig. 5). In May 2019, *Brevundimonas* (genus)*, Nocardioides* (genus)*, Olivibacter* (genus), *Paenibacillus* (genus), and Pedosphaeraceae (family) were all decreased in abundance in high severity soils relative to low severity soils (Fig. 5). In November 2020, all DA ASVs were increased in high severity soils relative to low severity soils (*Domibacillus* [genus]*, RB41* [genus], and Sporichthyaceae [family]), whereas the 10 DA ASVs identified in January 2023 were less abundant in high severity sites vs low severity sites (Fig. 5; File S3). October 2021 yielded 62 DA ASVs, the most among all time points analyzed and the majority of which were lower in abundance in the high severity soils. Altogether, these data highlighted greater stability in the low severity burned soils overtime.

### Low and high severity burn sites show differential bacterial functional capacity over time

**Fig 6:**
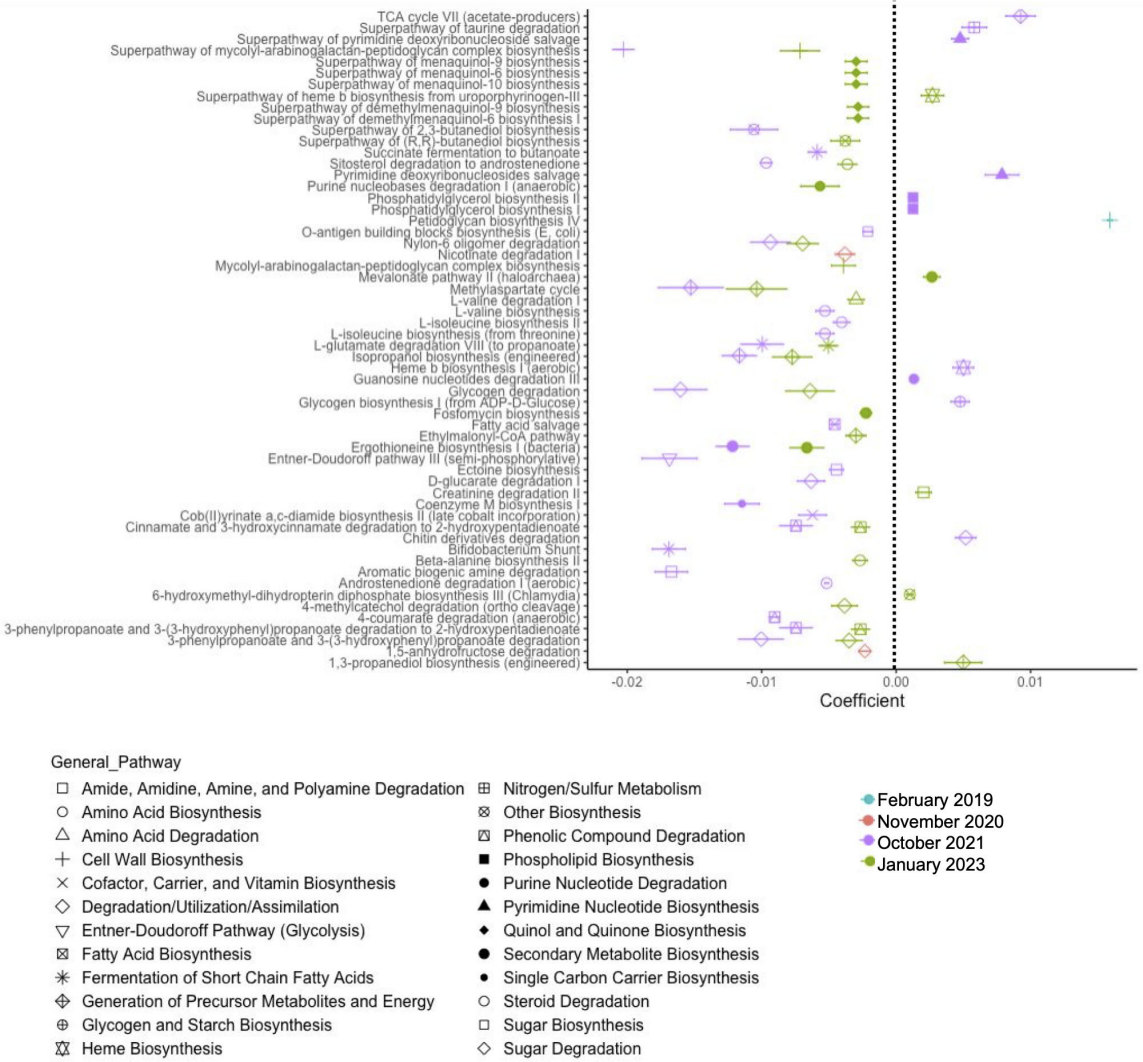
Differentially abundant predicted metabolic pathways in high severity burn sites relative to low severity burn sites. Coefficient is equivalent to the effect size and is a measure of the difference in abundance of the pathway in the high severity burned soil relative to the low severity burned soil. February 2019 (*n* = 4, 2 low and 2 high), November 2020 (*n* = 9, 5 low and 4 high), October 2021 (*n* = 5, 2 low and 3 high), and January 2023 (*n* = 14, 8 low and 6 high). *q* (Benjamin-Hochberg adjustment) value threshold <0.01.

We used PICRUSt2 (30) with the Kyoto Encyclopedia of Genes and Genomes (KEGG) (31–33) and MetaCyc (34) databases to predict the soil functional metagenome in the low and high severity burn sites over time. PICRUSt2 identified 7,503 KEGG Orthologs (KOs) correlated with the soil 16S rRNA gene sequence data and 427 MetaCyc pathways. We used MaAsLin2 to identify DA KOs (File S4) and Metacyc pathways between the low and high severity sites each year (Fig. 6; File S5), considering months (Fig. 6; Files S4 and S5) and across time points (similar to the compositional analyses, *q* < 0.25, Files S2, S4 and S5) (35). We note a decrease in bacterial KO abundance in high severity burn sites relative to low severity sites in all of the time points following the Woolsey fire (except for July 2021 which did not result in any DA KOs, File S4). All DA KOs in high relative to low severity burned sites were decreased in February and May 2019, 1,051 out of 1,353 DA KOs are decreased in November 2020, 917 out of 1,559 DA KOs were decreased in October 2021, and 508 out of 575 DA KOs are decreased in 2023 (File S4).

The KO analysis highlights decreased bacterial functionality in the high severity soil, and we further characterized this pattern through DA analysis of MetaCyc pathways derived from PICRUSt2. 1,381 out of 2,202 pathways were decreased in high severity burned soil, while 943 out of 1,209 pathways were decreased in low severity burned soil in May 2019, November 2020, October 2021, and January 2023 relative to February 2019 (File S5). We include detailed descriptors along with more generalized pathways for all DA MetaCyc pathways identified in high severity burned soil relative to low severity burned soil at a *q* value threshold of 0.1 (Fig. 6). No DA pathways were identified in May 2019 or July 2021. In February 2019, peptidoglycan synthesis IV was the only DA pathway identified, and it was increased in high severity soil relative to low severity soil (Fig. 6; File S5). November 2020 yielded two DA pathways, nicotinate degradation I and 1,5-anhydrofructose degradation, and both were decreased in high severity soil relative to low severity soil (Fig. 6; File S5). October 2021 and January 2023 yielded the most DA pathways between the two burn sites. In October 2021, 37 DA pathways were identified and 27 of them were decreased in high severity burned soil (Fig. 6). Of the 10 pathways that were increased in high severity burned soil in October 2021, eight were biosynthesis pathways (phosphatidylglycerol biosynthesis I & II, glycogen biosynthesis, superpathway of pyrimidine deoxyribonucleotide salvage, pyrimidine deoxyribonucleosides salvage, and heme B biosynthesis, and TCA cycle VII) while three were degradative (guanosine nucleotides degradation III, chitin derivatives degradation, and superpathway of taurine degradation) pathways (Fig. 6; File S5). In January 2023, 29 DA pathways were identified, and 24 are decreased in the high severity soil relative to the low severity soil. Of the five pathways that were increased in the high severity soil, four were classified into biosynthesis pathways (1,3-propanediol biosynthesis superpathway of heme b biosynthesis from uroporphyrinogen-III, mevalonate pathway II, 6-hydroxymethyl-dihydropterin diphosphate biosynthesis III) and 1 was degradative (creatinine degradation II, File S5; Fig. 6). These assessments of soil microbial functional capacity highlight further the divergence of the low and high severity burned soil bacteriome over time, with decreased metabolic capacity (suggested by the majority of DA pathways being decreased in abundance) of the soil bacteriome in the high severity burned soil particularly in October 2021 and January 2023.

### Decreased wildfire severity due to pre-fire VTC modifies relationships between soil bacterial taxa and their functional pathways

We conducted Spearman’s rank correlations (adjusted *P* value threshold < 0.05) between DA bacterial taxa and alpha diversity, and functional pathways in February 2019, November 2020, October 2021, and January 2023 including all soil samples (both high and low severity burned samples) and after stratification by burn severity to determine whether decreased burn severity due to vegetation thinning modifies any of the relationships between taxa/diversity and pathways. In November 2020, *Domibacillus* and RB41 were both negatively associated with 1, 5 anhydrofructose degradation and nicotinate degradation I (Table 1). 1, 5 anhydrofructose degradation was also positively associated with Shannon diversity across all samples and with Chao1 and Shannon diversity metrics in high severity burned samples, suggesting that increased burn severity modifies the association of this pathway with soil bacterial alpha diversity (Table 1). In October 2021, Chao1 and Shannon diversity were positively correlated with superpathway of 2,3-butanediol biosynthesis and aromatic biogenic amine degradation and negatively correlated with glycogen biosynthesis I, methylaspartate cycle, chitin derivatives degradation, pyrimidine deoxyribonucleosides salvage, the superpathway of pyrimidine deoxyribonucleoside salvage, and TCA cycle VII. *Segetibacter* was positively associated with chitin derivatives degradation across all samples, and this association remains only in high severity burned soils after stratification (Table 2). *Burkholderia-Caballeronia-Paraburkholderia*, *67-14*, *Chthoniobacteri*, Gaiellales (bacterial order), *Segetibacter*, *Actinoallomurus*, *Ellin6067*, *Chitinophaga*, *Bradyrhizobium*, and *Aeromicrobium* were all positively associated with both superpathway of mycolyl-arabinogalactan-peptidoglycan complex biosynthesis and sitosterol degradation to androstenedione (Table 2) across all soil samples. *Nocardioides*, *Solirubrobacter*, and Sphingomonadaceae (bacterial family) were associated (positively and negatively) with numerous pathways in the high severity burned soils only (Table 2). These results suggest higher burn severity modifies relationships between bacterial taxa and overall bacterial diversity and functional genes. In other words, these associations were not present in soils that experienced low burn severity due to pre-fire VTC, further emphasizing the divergence of the two soil sites over time.

**TABLE 1:** Significant Spearman correlations between bacterial diversity and DA taxa and bacterial functional pathways in 2020

Taxa or diversity measure	Pathway(s)	Rho	Adjusted *P* value (Holm’s)	Group (total, high, or low)
*Domibacillus* (ASV 1524)	1, 5 andhydrofructose degradation (PWY-6992)	−0.86	0.01	Total
	Nicotinate degradation I (PWY-722)	−0.75	0.04	Total
RB41 (ASV 3352)	1, 5 andhydrofructose degradation (PWY-6992)	−0.71	0.04	Total
	Nicotinate degradation I (PWY-722)	−0.83	0.02	Total
Chao1 diversity	1, 5 andhydrofructose degradation (PWY-6992)	0.78 1	0.05 0	Total High
Shannon diversity	1, 5 andhydrofructose degradation (PWY-6992)	1	0	High

**TABLE 2:** Significant spearman correlations between bacterial diversity and DA taxa and bacterial functional pathways in 2021

Taxa or diversity measure	Pathway	Rho	Adjusted *P* value (Holm’s)	Group (total, high, or low)
*Segetibacter* (ASV 3055)	Chitin derivatives degradation (PWY-6906)	1	0	Total
		1	0	High
	Glycogen Biosynthesis I (GLYCOGENSYNTH-PWY)	1	0	High
	Superpathway of 2,3-butanediol biosynthesis (PWY-6396)	−1	0	High
	Methylaspartate cycle (PWY-6728)	1	0	High
	Pyrimidine deoxyribonucleosides salvage (PWY-7199)	1	0	High
	Superpathway of pyrimidine deoxyribonucleoside salvage (PWY-7200)	1	0	High
	TCA cycle VII (acetate-producers, PWY-7254)	1	0	High
	Aromatic biogenic amine degradation (PWY-7431)	−1	0	High
*67-14* (ASV 588)	Superpathway of mycolyl-arabinogalactan-peptidoglycan complex biosynthesis (PWY-6404)	1	2.8e−21	Total
	Sitosterol degradation to androstenedione (PWY-6948)	1	2.8e−21	Total
*Burkholderia-* *Caballeronia-* *Paraburkholderia* (ASV 1034)	Superpathway of mycolyl-arabinogalactan-peptidoglycan complex biosynthesis (PWY-6404)	1	2.8e−21	Total
	Sitosterol degradation to androstenedione (PWY-6948)	1	2.8e−21	Total
*Chthoniobacter* (ASV 1829)	Superpathway of mycolyl-arabinogalactan-peptidoglycan complex biosynthesis (PWY-6404)	1	2.8e−21	Total
	Sitosterol degradation to androstenedione (PWY-6948)	1	2.8e−21	Total
Gaiellales (ASV 1861)	Superpathway of mycolyl-arabinogalactan-peptidoglycan complex biosynthesis (PWY-6404)	1	2.8e−21	Total
	Sitosterol degradation to androstenedione (PWY-6948)	1	2.8e−21	Total
*Segetibacter* (ASV 2632)	Superpathway of mycolyl-arabinogalactan-peptidoglycan complex biosynthesis (PWY-6404)	1	2.8e−21	Total
	Sitosterol degradation to androstenedione (PWY-6948)	1	2.8e−21	Total
*Actinoallomurus* (ASV 5523)	Superpathway of mycolyl-arabinogalactan-peptidoglycan complex biosynthesis (PWY-6404)	1	2.8e−21	Total
	Sitosterol degradation to androstenedione (PWY-6948)	1	2.8e−21	Total
*Ellin6067* (ASV 5688)	Superpathway of mycolyl-arabinogalactan-peptidoglycan complex biosynthesis (PWY-6404)	1	2.8e−21	Total
	Sitosterol degradation to androstenedione (PWY-6948)	1	2.8e−21	Total
*Chitinophaga* (ASV 6248)	Superpathway of mycolyl-arabinogalactan-peptidoglycan complex biosynthesis (PWY-6404)	1	2.8e−21	Total
	Sitosterol degradation to androstenedione (PWY-6948)	1	2.8e−21	Total
*Bradyrhizobium* (ASV 6404)	Superpathway of mycolyl-arabinogalactan-peptidoglycan complex biosynthesis (PWY-6404)	1	2.8e−21	Total
	Sitosterol degradation to androstenedione (PWY-6948)	1	2.8e−21	Total
*Aeromicrobium* (ASV 7572)	Superpathway of mycolyl-arabinogalactan-peptidoglycan complex biosynthesis (PWY-6404)	1	2.8e−21	Total
	Sitosterol degradation to androstenedione (PWY-6948)	1	2.8e−21	Total
*Nocardioides* (ASV 3475)	O-antigen building blocks biosynthesis (*E. coli*, OANTIGEN-PWY)	−1	0	High
	Ectoine biosynthesis (P101-PWY)	1	0	High
	*Bifidobacterium* Shunt (P124-PWY)	−1	0	High
	Coenzyme M biosynthesis I (P261-PWY)	−1	0	High
	Nylon-6 oligomer degradation (P621-PWY)	−1	0	High
	Entner-Doudoroff pathway III (semi-phosphorylative, PWY-2221)	−1	0	High
	L-glutamate degradation VIII (to propanoate, PWY-5088)	−1	0	High
	Succinate fermentation to butanoate (PWY-5677)	1	0	High
	Glycogen degradation (PWY-5941)	−1	0	High
	Isopropanol biosynthesis (engineered, PWY-5941)	−1	0	High
	Androstenedione degradation I (aerobic, PWY-6944)	−1	0	High
	Ergothioneine biosynthesis I (bacteria, PWY-7255)	−1	0	High
	3-Phenylpropanoate and 3-(3-hydroxyphenyl)propanoate degradation (PWY0-1277)	−1	0	High
	Phosphatidylglycerol biosynthesis I (PWY4FS-7)	−1	0	High
	Phosphatidylglycerol biosynthesis II (PWY4FS-8)	−1	0	High
*Solirubrobacter* (ASV 4940)	D-glucarate degradation I (GLUCARDEG-PWY)	−1	0	High
	3-Phenylpropanoate and 3-(3-hydroxyphenyl)propanoate degradation to 2-hydroxypentadienoate (HCAMHPDEG-PWY)	−1	0	High
	Heme b biosynthesis I (aerobic, HEME-BIOSYNTHESIS-II)	1	0	High
	L-isoleucine biosynthesis (from threonine, ILEUSYN-PWY)	−1	0	High
	Superpathway of taurine degradation (PWY-1541)	−1	0	High
	L-isoleucine biosynthesis II (PWY-5101)	−1	0	High
	Guanosine nucleotides degradation III (PWY-6608)	−1	0	High
	Cinnamate and 3-hydroxycinnamate degradation to 2-hydroxypentadienoate (PWY-6691)	−1	0	High
	4-Coumarate degradation (anaerobic, PWY-7046)	−1	0	High
	Fatty acid salvage (PWY-7094)	−1	0	High
	Cob(II)yrinate a,c-diamide biosynthesis II (late cobalt incorporation, PWY-7376)	−1	0	High
	L-valine biosynthesis (VALSYN-PWY)	−1	0	High
Sphingomonadaceae (*ASV 656*)	D-glucarate degradation I (GLUCARDEG-PWY)	1	0	High
	3-Phenylpropanoate and 3-(3-hydroxyphenyl)propanoate degradation to 2-hydroxypentadienoate (HCAMHPDEG-PWY)	1	0	High
	Heme b biosynthesis I (aerobic, HEME-BIOSYNTHESIS-II)	1	0	High
	L-isoleucine biosynthesis (from threonine, ILEUSYN-PWY)	1	0	High
	Superpathway of taurine degradation (PWY-1541)	1	0	High
	L-isoleucine biosynthesis II (PWY-5101)	1	0	High
	Guanosine nucleotides degradation III (PWY-6608)	1	0	High
	Cinnamate and 3-hydroxycinnamate degradation to 2-hydroxypentadienoate (PWY-6691)	1	0	High
	4-Coumarate degradation (anaerobic, PWY-7046)	1	0	High
	Fatty acid salvage (PWY-7094)	1	0	High
	Cob(II)yrinate a,c-diamide biosynthesis II (late cobalt incorporation, PWY-7376)	1	0	High
	L-valine biosynthesis (VALSYN-PWY)	1	0	High
Chao1 diversity	Glycogen Biosynthesis I (GLYCOGENSYNTH-PWY)	−1	0	High
	Superpathway of 2,3-butanediol biosynthesis (PWY-6396)	1	0	High
	Methylaspartate cycle (PWY-6728)	−1	0	High
	Chitin derivatives degradation (PWY-6906)	−1	0	High
	Pyrimidine deoxyribonucleosides salvage (PWY-7199)	−1	0	High
	Superpathway of pyrimidine deoxyribonucleoside salvage (PWY-7200)	−1	0	High
	TCA cycle VII (acetate-producers, PWY-7254)	−1	0	High
	Aromatic biogenic amine degradation (PWY-7431)	1	0	High
Shannon diversity	Glycogen Biosynthesis I (GLYCOGENSYNTH-PWY)	−1	0	High
	Superpathway of 2,3-butanediol biosynthesis (PWY-6396)	1	0	High
	Methylaspartate cycle (PWY-6728)	−1	0	High
	Chitin derivatives degradation (PWY-6906)	−1	0	High
	Pyrimidine deoxyribonucleosides salvage (PWY-7199)	−1	0	High
	Superpathway of pyrimidine deoxyribonucleoside salvage (PWY-7200)	−1	0	High
	TCA cycle VII (acetate-producers, PWY-7254)	−1	0	High
	Aromatic biogenic amine degradation (PWY-7431)	1	0	High

## DISCUSSION

The objective of this study was to assess the effect of VTC induced by chaparral shrub thinning prior to wildfire on the longitudinal response of the soil microenvironment to the Woolsey wildfire (November 2018). Our findings support our hypothesis that lower vegetation burn severity due to pre-fire vegetation thinning and VTC longitudinally alter soil chemistry as well as bacterial postfire recovery and postfire succession. Specifically, low severity burned soils experienced lower percent of organic matter, pH, and N, P, and K concentrations in 2019 (Fig. 2). PCoA highlights divergence of the high and low severity soils by January 2023 (approximately 4.25 years after the Woolsey fire, Fig. 3D). Bacterial abundances were more stable over time in low severity burned soils as compared with high severity soils (Fig. 4 and 5). Furthermore, high severity soils showed colonization with more pyrophilous taxa in the early months following the fire (e.g., Bacillaceae spp.), with an increased abundance of pyrophilous taxa (Xanthobacteraceae spp., *Soirubrobacter* spp.) in low severity soils in the later years after the fire (Fig. 4 and 5). The two soil types also differed in bacterial functional capacity. Longitudinally, the majority of pathways were decreased in abundance in high severity soils, highlighting a pattern of potential downregulation of the bacteriome (Fig. 6). The peptidoglycan synthesis IV pathway was increased in high severity soils relative to low severity soils in 2019. In the later years following the fire (October 2021 and January 2023), of the few pathways that were increased in the high severity soil these were primarily biosynthesis pathways (e.g., heme B biosynthesis in October 2021 and January 2023 and glycogen biosynthesis in October 2021). Chitin degradation and guanosine nucleotide degradation (October 2021) and creatinine degradation (January 2023) were among the degradative pathways that were increased in high severity relative to low severity soil in the later years. Finally, increased fire severity modified correlations between bacteria and functional pathways in November 2020 and October 2021, further emphasizing the divergence of these soils over time. These findings represent implications for fire management strategies, which induce VTC prior to wildfire. Our work suggests the impact of these strategies not only change the vegetative ecology, but also the microbial ecology of the soil, which results in altered microbial succession after wildfire.

Soil respiration typically increases immediately following wildfire as microbes in the soil increase their metabolic activity to decompose burned organic matter (36). In our study, both high and low severity burned soils showed decreased respiration in 2020 relative to 2019, with high severity soils showing sustained decreases in respiration in both 2020 and 2021 relative to 2019 (Fig. 1A). This suggests an increase in respiration (consistent with the literature [36]) initially after the fire and a gradual decrease in metabolic activity of soil microbes as time passes. Our group reports higher respiration rates in high severity burned soil in 2019 following the Woolsey fire (37). However, we did not identify significant differences in soil respiration across the two burned sites after post-hoc analysis by year and burn severity (Fig. 1). Bacterial metabolic capacity was decreased in both soils (low and high) in 2020 and 2021 (relative to February 2019), which correlates with decreased respiration over time (File S5). Interestingly in October 2021, degradation of chitin derivatives was increased in high severity soil relative to low severity soil. Fungal biomass was reported to be positively associated with soil organic decomposition (38). Since high severity burned soils showed decreased bacterial diversity and metabolic capacity in November 2020 and October 2021 relative to low severity soils, this suggests the majority of the CO_2_ production measured could be due to increased respiration by the mycobiome. In support of this hypothesis, our group has extensive supporting evidence of fungal growth on chaparral plants in this region following wildfire (39, 40).

Both soils show decreased percent organic matter in 2021 relative to 2019 (Fig. 2A). This result suggests increased microbial decomposition of organic matter over time. The differential fuel load due to more woody chaparral species at the high severity burned site presumably accounts for the increased organic matter. Relating these findings to bacterial functional capacity, we identified one bacterial pathway in February 2019 that was increased in high severity soils relative to low severity soils, peptidoglycan biosynthesis IV (PWY-6471). This pathway suggests growth of bacteria in February 2019, which would correspond to increased consumption of organic matter in the early months following the Woolsey fire. In high severity soil relative to low severity soils, we identified two pathways in November 2020, nicotinate degradation I and 1,5-anhydrofructose degradation, both of which were decreased in the high severity burned soils relative to the low severity burned soils (Fig. 6). In October 2021, there were 37 DA pathways identified and 27 of them were decreased in high severity burned soil relative to low severity burned soil (Fig. 6). Interestingly, one of the pathways decreased in high severity soil in January 2023 was the superpathway of mycolyl-arabinogalactan-peptidoglycan complex biosynthesis, suggesting bacterial taxa are not growing as rapidly as those in low severity burned soils (Fig. 6). These decreased pathway findings suggest decreased bacterial decomposition of soil organic matter in November 2020, October 2021, and January 2023 in high severity soil relative to low severity soils, which may account for the lack of differences in % organics in 2021 (Fig. 2A).

Low severity burned soils also showed decreased N, P, and K availability and pH in 2019 compared to high severity burned soils (Fig. 2B through E) similar to previous studies (41, 42). Many of the DA bacterial taxa between the high and low severity burned sites are nitrogen fixers (Bacillaceae, *Domibacillus, Beijerinckiaceae,* Fig. 5) (43). *Bacillus* spp. are also P and K solubilizers, emphasizing the influence of N, P, and K availability on the increased abundances of these bacterial taxa in the high severity burned soils (43–45). Additionally, the increased abundance of the peptidoglycan synthesis pathway in 2019 highlights increased growth of taxa in the high severity burned soils and the need for nitrogen and carbon (found in the increased organic matter) for cell wall production (Fig. 6). Increased soil nutrients were reported to be associated with burn severity (42). However, in our study, we cannot differentiate between burn severity and VTC prior to fire as possible influences on soil chemistry.

Significant differences in bacterial richness (based on the Chao1 index) were only observed in the high severity soils from May 2019 to January 2023 (Fig. 3F and H). This suggests that the high severity burn site may be more prone to longitudinal shifts in bacterial richness than the low severity, pre-fire vegetation thinned burn site. Burn severity has been shown to alter bacterial composition of the soil (46), and our analysis of beta diversity and relative abundance supports this. We identified increased divergence of the high and low severity soils over time, with the two sites diverging in global bacterial composition (as seen via PCoA based on unweighted UniFrac distances) in January 2023 (Fig. 3D). In support of this beta diversity divergence, high severity soils displayed an increased abundance of the phylum, Bacteroidetes (consistent with previous studies [47]), in January 2023 (Fig. 4C). Furthermore, the majority of DA bacterial taxa between the low and high severity sites were identified in October 2021 and January 2023 (Fig. 5). While the soils diverge in the later years following the Woolsey fire, in the early months (February and May 2019), we also note phylum and genus level differences between the sites, specifically related to abundances of pyrophilous taxa.

Both high and low severity burned soils showed increases in Proteobacteria in February 2019, emphasizing colonization with pyrophilous taxa at both sites shortly after the fire (Fig. 4A through D), which is consistent with previous studies (22, 24). Though both sites showed increased abundances of fire-primed microbes shortly after the wildfire, we identified an increase in pyrophilous taxa in high severity burned soils relative to low severity burned soils in the earlier time points following the Woolsey fire. Specifically, high severity burned soils showed increased abundances of Bacillaceae (February 2019), *Domibacillus* (2020), and *RB41* (2020) (Fig. 4 and 5). Bacillaceae spp. can form heat-resistant endospores, which are typical following fire (18). At later time points (October 2021 and January 2023), high severity burned soils show increased abundances of *Noviherbaspirillum*, *Rhizobales*, and Beijerinckiaceae, relative to low severity burned soils (Fig. 5). Some of these taxa (*Noviherbaspirillum, Domibacillus,* and *RB41*) were reported as increased in burned MTE soils by Pulido et al., and our study suggests their elevated abundances are associated with increased burn severity due to lack of pre-fire vegetation thinning (22). Bacteriome compositional analysis also suggests dynamic bacterial succession patterns in high severity burned soils in the years following the Woolsey fire. Low severity burned soils were more compositionally consistent, while high severity burned soils changed more rapidly in bacterial abundance over time (Fig. 4 and 5). In our study, low severity burned soils were characterized by increased *Massilia*, *Conexibacter*, *Solirubrobacter*, *Segetibacter*, and Xanthobacteraceae relative to high severity burned soils in October 2021 and January 2023. Interestingly, many of these taxa are pyrophilous, suggesting that low severity wildfires instigated by vegetation thinning can result in sustained colonization (at least for 4.25 years) of soil with bacterial taxa primed for wildfire. This is further supported by the expanding compositional differences between the two sites (increased taxonomic differences in October 2021 and January 2023) and their divergence in beta diversity in January 2023 (Fig. 3 to 5). To our knowledge, there is only one other study that has analyzed the soil microbiome following the Woolsey wildfire (24). Though this study did not sample high and low severity burned sites, they did sample study sites characterized by either native or non-native chaparral plants pre- and post-wildfire and found that the plant species present heavily influenced the soil bacteriome composition (24). In our study, the low severity burn site displays VTC prior to fire, with more invasive grasses and forbs colonizing this site due to pre-fire woody chaparral species reduction as a fire mitigation strategy. We cannot distinguish between the effects of the vegetation type and burn severity. However, in line with Cox et al., this work provides compelling evidence that VTC prior to fire and associated with lower burn severity begets altered bacterial successional dynamics and recovery in the chaparral soil of the Santa Monica Mountains.

As mentioned previously, we also identified decreased functional capacity of the bacteriome, particularly in the later years following the Woolsey fire (Fig. 6). We correlated differential functional pathways with bacterial taxa and diversity and identified positive correlations between Shannon and Chao1 diversity and 1,5 anhydrofructose degradation in November 2020. These associations remained only in high severity burned soils after stratification (Tables 1 and 2). This suggests that increased bacterial diversity in the high severity burned soil is associated with degradation of glycogen and starch. In October 2021, many associations between diversity and pathways were negatively correlated (glycogen biosynthesis, chitin derivatives degradation, TCA cycle VII, etc.), which as expected suggests that as bacterial richness and distribution increases, the functionality of the bacteriome shifts. This analysis also emphasizes the functional roles of some pyrophilous taxa (*Domibacillus, RB41, Segetibacter*, *Nocardioides,* Tables 1 and 2). For example, *Segetibacter* is associated with chitin derivatives degradation, glycogen biosynthesis, and pyrimidine biosynthesis, all of which were increased in high severity soils (Fig. 6). Some of the functional pathways associated with these taxa are potentially instigated by bacteria as a protective strategy to cope with changes in soil chemistry. For example, *Nocardioides* was positively associated with ectoine biosynthesis, a stress protectant produced by microbes to cope with changes in osmotic pressure and to protect against DNA damage (17, 48). This analysis also highlights roles of high severity soil bacterial taxa (*Nocardioides*, *Segetibacter, Domibacillus, RB41*) in N and C cycling (specific pathways: L-Isoleucine and L-Valine biosynthesis, glycogen biosynthesis, succinate fermentation, sitosterol degradation, anhydrofructose degradation, etc.), functions of key importance to the natural functionality of our ecosystems (Tables 1 and 2) (18). It is possible that we are able to distinguish specific bacterial roles in high severity burned soils because these soils experience more dynamic shifts in bacterial abundance and metabolic capacity over time, as compared with low severity soil bacteriomes, which are more stable overtime. Thus, this analysis provides further evidence to support altered soil bacterial successional dynamics when fire mitigation strategies change the vegetation and corresponding burn severity from the more natural wildfire experience of the woody chaparral in the SMMs.

Our study is the first to analyze the effect of vegetation thinning induced VTC and associated burn severity on the soil bacteriome in a MTE. We integrated genomic and non-genomic (measurements of soil respiration and nutrients) assessments to characterize the soil microbiome. Applying PICRUSt2 to our 16S rRNA gene sequencing data also provided us with valuable functional information about the soil bacteriome without the need for additional DNA sequencing. Our study also has limitations. We do not have unburned soil samples from the high and low severity sites, so we cannot differentiate between burn severity and vegetation type in our analysis of the soil microenvironment. However, we provide evidence for decreased burn severity due to vegetation thinning. To our knowledge, there are no studies that have addressed the effect of these highly intertwined ecological aspects of chaparral fire ecology on the soil bacteriome, highlighting a novel addition to the literature that has not been previously addressed. Our sample sizes in the earlier time points (particularly in February and May 2019 where we only have two soil samples for high severity and two soil samples for low severity per time point) are small. This limits our ability to effectively compare high and low severity burned soils during those time points. We also have limited parallel time points for soil respiration, nutrient availability, and microbiome analysis. Though the sites of these analyses overlap, the precise time points do not consistently overlap. This particularly limits our ability to conduct associative analyses between specific bacterial taxa, nutrients, CO_2_ production, and functional pathways. Finally, our study was designed to focus on the soil bacteriome, but our data highlight the necessity to characterize additional microbial species in these burn sites. We plan to conduct metagenomic or fungal microbiome targeted sequencing on soil samples in the future to test our hypotheses about fungal contributions to soil respiration.

In conclusion, our findings support our hypothesis that lower vegetation burn severity due to pre-fire vegetation thinning and type conversion would result in longitudinal alterations to soil chemistry, bacterial recovery, and bacterial succession. Low severity soils displayed lower nutrient availability, pH, and percent organic matter in 2019. PCoA highlights community compositional (beta diversity) divergence of low and high severity soils by January 2023. Analysis of bacterial relative abundance suggests this divergence is guided by a prolonged presence of pyrophilous taxa in low severity soil. These compositional differences between high and low severity soils are accompanied by increased bacterial cell wall production in February 2019 in high severity soils relative to low severity soils. In November 2020, October 2021, and January 2023, low severity soils show increased metabolic capacity relative to high severity soils. Altogether, these findings suggest that VTC induced by pre-fire vegetation thinning decreases the severity of the wildfire and results in altered soil chemistry and prolonged changes in bacterial composition. Next steps in this work involve continued assessments of soil chemistry, respiration, and microbial composition and function over time, which will allow us to determine how long these changes in soil bacterial composition last after wildfire. We will also be able to capture the microbial signature of soil prior to wildfire, particularly as these sites recently burned again during the Franklin fire in December 2024. Our findings represent profound implications for fire management strategies in the SMMs, which accompany the increasing WUI and increased fire return interval (~6 years between Woolsey and Franklin) experienced by this region. VTC introduced by pre-fire vegetation thinning to protect structures from wildfire is altering the plant ecology in this region, and this work suggests this may be accompanied by longitudinal changes to the soil microbiome. Without proper preservation of the natural chaparral landscape, these mitigation strategies are at risk of creating a feedback loop wherein chaparral VTC begets altered soil bacterial composition and function, which begets continued and potentially irreversible VTC. Further studies in this area, involving controlled soil experiments, will determine whether it may also be possible to modify VTC with bacterial supplementation of soil, supporting reestablishment of the natural chaparral landscape which continues to be under threat due to anthropogenic climate change.

## MATERIALS AND METHODS

### Experimental design

This study was conducted on the natural landscape of Pepperdine University’s campus in Malibu, CA. This landscape represents an MTE set in the SMM of southern California. We sampled roughly 100 mm of soil from the organic horizon (O) and upper mineral (A) regions with a sterilized 50 mL conical tube from two study sites located adjacent to to each other at an elevation of 228 m (34°02′ 51″ N, 118°42′ 49²″ W). The two sites both burned during the Woolsey fire (which burned 39,234 ha in November 2018), but vegetation fuel load of the low severity burn site was thinned prior to the wildfire by 80% to form a 100 m wide fuel break as defensive space to protect campus structures during wildfire. Thus prior to the fire, the low severity burn site was dominated by invasive grasses and forbs, which burn quickly and typically at a cooler temperature, while the high severity burn site represents the more natural landscape and shrub-dominated composition of the SMMs, which should burn hotter and slower.

### Burn severity

Burn severity was estimated by measuring the diameter of the smallest burned twig on five adult *Ceanothus spinosus* individuals per severity category, with three burned branches sampled per individual. Using vernier calipers, we measured the diameter of the smallest clearly charred twig within each branch that retained visible ash from the Woolsey Fire. For each twig, two perpendicular diameter measurements were taken and averaged to obtain a mean diameter. A 4.34-fold larger diameter of stems remained in the higher severity burn site relative to the low severity burn site. This method is based on the assumption that lower severity fires burn at lower temperatures and for shorter durations, leaving behind smaller twigs, while higher severity fires burn hotter and longer, consuming smaller twigs and leaving only larger ones (adapted from reference 49).

### Soil respiration

We used portable gas-exchange systems (Model LI-6800, LI-COR Environmental, Lincoln, NE, USA), connected to a soil CO_2_ flux chamber (Model # Li-6800-09), equipped with a Stevens HydraProbe (Model # 900-19016) to monitor soil moisture content and soil temperature during respiration measurements. Soil respiration was estimated in terms of CO_2_ efflux from the soil surface in units of µmol CO_2_ m^−2^ s^−1^. PVC collars, open at both ends, 200 mm in diameter and 114 mm high, were inserted ~74 mm deep in soil surfaces, leaving 20–30 mm of inside collar-height above the soil surface, facilitating gasket-connection to the CO_2_ flux chamber. Twelve collars at each burn site (high severity burn site plus low severity burn site = 24 collars total) were permanently installed allowing repeated measurements throughout the experiment. First respiration measurements were made at least 1 week after initial collar installation to ensure soil gas distribution returned to equilibrium prior to data collection. Final *n* numbers for measurements: 2019 high *n* = 62 (May *n* = 11, June 4 *n* = 12, June 11 *n* = 9, June 17 *n* = 12, June 28 *n* = 12, July *n* = 6), low *n* = 72 (*n* = 12 for all time points), 2020 high *n* = 39, 2020 low *n* = 33, 2021 high *n* = 78 (February *n* = 39, March *n* = 39), 2021 low *n* = 66 (February *n* = 22, March *n* = 33). Further details regarding respiration measurements can be found in File S2.

### Soil nutrients

Soil samples for measurement of percent organics, mineral nutrients (N, P, and K), and pH were extracted at a depth range of 20–100 mm to coincide with surface measurement of soil respiration and soil bacteriome analyses. Sample fresh mass typically ranged between 200 and 300 g. Surface litter composed of leaf and twig fragments generally occupied the first 20 mm of depth and was, thus, excluded from nutrient sampling. We selected 24 soil sampling sites 15–30 mm distant from the outer edge of each of our CO_2_ efflux collars, 12 at low severity burn sites and 12 at high severity burn sites. Final *n* numbers: 2019 high severity *n* = 10, 2019 low severity *n* = 11. 2021 high severity *n* = 12, 2021 low severity *n* = 12. For soil extraction, we used an Oakfield soil sampler (Model DB3, Oakfield, WI, USA). Soil samples were screened for roots and deleted from soil samples when detected. Samples were returned to the lab within 30 min of collection, immediately placed in a drying oven at 40°C for at least 48 h to promote air drying and then packaged for shipment to Utah State University’s Analytical Labs, in Logan UT, USA. Analyses included % organic matter, pH, nitrate-nitrogen in mg/kg, potassium in mg/kg, and phosphorus in mg/kg.

### Microbiome analysis

#### Soil sample collection

Soil samples for microbiome analysis were collected at a depth of 20–100 mm in sterile 50 mL conical tubes. Sample fresh mass typically ranged between 70 and 80 g. Samples were collected beginning in February 2019 (*n*_high_ = 2, *n*_low_ = 2), following the Woolsey wildfire that burned 39,234 ha in November 2018. Samples were subsequently collected from these study sites in May 2019 (*n*_high_ = 2, *n*_low_ = 2), November 2020 (*n*_high_ = 4, *n*_low_ = 5), July 2021 (*n*_high_ = 2, *n*_low_ = 3), October 2021 (*n*_high_ = 3, *n*_low_ = 2), and January 2023 (*n*_high_ = 6, *n*_low_ = 8) (File S2; Table S1). Samples were screened for roots, and roots were deleted from soil samples when detected. Samples were returned to the lab within 30 min of collection and were stored at −80℃ prior to subsequent DNA extraction.

#### DNA isolation

Metagenomic DNA was extracted from soil samples using the Qiagen DNeasy PowerSoil Pro kit (Qiagen, Hilden, Germany). Approximately 0.25 grams of frozen soil was used for DNA isolation per the manufacturer’s instructions. Soil samples were homogenized in bead tubes in the FastPrep homogenizer (MP Biochemicals, Irvine, CA) for 2 min (alternative to 10 minutes of vortexing). In addition to isolating DNA from the soil samples, we also isolated DNA from two positive controls (ZymoBIOMICS microbial community standard [Catalog No. D6300]) and five negative extraction controls. Positive and negative controls were subjected to the same DNA isolation procedure, library preparation, and sequencing. Genus abundances from the positive control samples can be found in the supplementary materials (File S2).

#### Library preparation and sequencing

Extracted DNA from soil samples and positive and negative controls were submitted to the University of California Davis Host Microbe Systems Core Lab for library preparation and Illumina Miseq bidirectional sequencing (2 × 300 bp, Illumina, San Diego, CA, USA) of the V3-V4 regions of the 16S gene (primers: 319F and 806R).

### Statistical analysis

#### Analysis of respiration, pH, and nutrient data

We used the Shapiro-Wilk test for normality to determine if nutrients, pH, and respiration data sets were normally distributed. For nutrients and pH, pH was the only variable where the data were normally distributed. The respiration data were not normally distributed. Following tests for normality, we used ANOVA with post-hoc Tukey tests for normally distributed data and Kruskal-Wallis and post-hoc Dunn’s test for non-normally distributed data to compare data across time points and wildfire burn severity. *P* values reported for Fig. 1 and 2 are adjusted via Bonferroni adjustment and repeated letters on over groups in the figures represent non-significant differences between groups based on an adjusted *P* value < 0.05. All raw data sets and raw statistics can be found in supplementary materials (File S1).

#### Analysis of microbiome diversity

Microbiome sequence data preprocessing details and the raw ASV file with taxa classifications can be found in supplemental materials (Files S2 and S6). We used *Phyloseq* in R to analyze this data set for relative abundance of the top 100 ASVs and *MaAsLin2* to identify differentially abundant, relativized ASVs (across all ASVs) in high and low severity burned samples subsetted by year and across time points within the high and low severity burned groups (35, 50). For comparisons between high and low severity soils, the low severity soil is the reference group. For comparisons across time points, February 2019 is the reference group. Following the Shapiro-Wilk test for normality, Shannon diversity metrics were determined to not be normally distributed, and high and low severity samples were compared to each other and across years using Kruskal-Wallis with post-hoc Dunn’s tests (Bonferroni adjustment). Chao1 metrics were normally distributed (Shapiro-Wilk *P* > 0.05), and high and low severity samples were compared to each other and across years using ANOVA with post hoc Tukey tests. Beta diversity was assessed using PCoA (Principal Coordinates Analysis) based on weighted and unweighted UniFrac distances in high and low severity burned samples subsetted by year. We also subsetted by high and low severity soils and compared beta diversity over time. The betadispr function from the package *vegan* in R was used to determine the relative heterogeneity of the sample subsets (51). For all subsets, this test yielded a *P* > 0.05;, thus, PERMANOVA was applied to determine if the clustering of the samples on the PCoA was statistically significant based on burn severity or time (51). All plots were made in R using *ggplot2* (52).

#### Analysis of predicted microbiome function

We used PICRUSt2 under default settings to infer a profile of microbial functions from File S6 (the same ASV table used for relative abundance analyses) (30). PICRUSt2 (30) uses the Kyoto Encyclopedia of Genes and Genomes (KEGG) Database (31) to classify the inferred metagenome to KEGG orthologs (KOs) and the MetaCyc Database (34) to classify the metagenome into metabolic pathways. KOs and MetaCyc pathways can be found in supplementary materials (Files S7 and S8). Prior to analysis, we relativized these data sets. We then used ggpicrust2 (53) and MaAsLin2 (35) to identify differentially abundant KOs and metabolic pathways with a *q* value ≤ 0.25 (Benjamini-Hochberg adjustment) in high and low severity burned samples subsetted by year to compare severity or subsetted by severity across time points.

#### Correlations between microbial taxa, the predicted metagenome, and soil respiration and nutrients

We used the *psych* package in R to correlate (Spearman’s Rank correlation analysis) Shannon and Chao1 diversity metrics, differentially abundant taxa, and differentially abundant inferred metabolic pathways in the high- and low severity burned samples at each time point (*P* adjusted <0.05, Holm’s method) (54). Data used in these correlation analyses and corresponding results can be found in the supplementary materials (File S9).

## Data Availability

All Fastq files for 16S sequencing are deposited in the NCBI Sequence Read Archive (SRA) (accession number: PRJNA1176155). All R-code for preprocessing can be found in supplementary materials (File S10). All relevant datasets are available as additional files in the supplementary material. The authors encourage requests of any other datasets or information that would be helpful to those aiming to validate these findings.

## SUPPLEMENTAL MATERIAL

File S1 spectrum.00185-25-s0001.xlsx Metadata, respiration data and stats, Alpha div data and stats, nutrients and stats, and beta div stats.

File S2 spectrum.00185-25-s0002.docx Supplemental methods; Fig. S1 to S3.

File S3 spectrum.00185-25-s0003.xlsx Maaslin2 results for ASVs.

File S4 spectrum.00185-25-s0004.xlsx Maaslin2 results for KOs.

File S5 spectrum.00185-25-s0005.xlsx Maaslin2 results for pathways.

File S6 spectrum.00185-25-s0006.xlsx Raw ASV and taxa file.

File S7 spectrum.00185-25-s0007.txt Raw KO file.

File S8 spectrum.00185-25-s0008.txt Raw pathway file.

File S9 spectrum.00185-25-s0009.xlsx Correlation raw data and results where significant findings were identified.

File S10 spectrum.00185-25-s0010.docx R Code.

ASM does not own the copyrights to Supplemental Material that may be linked to, or accessed through, an article. The authors have granted ASM a non-exclusive, world-wide license to publish the Supplemental Material files. Please contact the corresponding author directly for reuse.
