# Supplementary material for: Effects of low wildfire burn severity due to pre-fire shrub thinning on the chaparral soil bacteriome in the Santa Monica Mountains of Southern California
Source: Microbiol Spectr. 2025 Jun 30;13(8):e00185-25. doi: 10.1128/spectrum.00185-25 (PMC12323652; doi:10.1128/spectrum.00185-25)
Supplement: File S2 — Supplemental methods; Fig. S1 to S3. [file spectrum.00185-25-s0002.docx]

**Effects of wildfire intensity on the chaparral soil bacteriome in the Santa Monica Mountains of southern California.**

Mariah Macias^1^, Mari R. Irving^1,2^, Katelyn M. Bandow^1^, Kaitlyn Kim^1^, Cecilia Heredia^1^, Courtney A. Hoskinson^1,3^, Nina R. Duchild^1^, Michael T. Nicholas^1^, Lindsey M. Marian^1^, Camille K. Sicangco^4^, Stephen D. Davis^1^, Helen I. Holmlund^1^, Leah T. Stiemsma^1^

1. Natural Science Division, Pepperdine University, Malibu, CA 90263 USA
2. Department of Ecology, Evolution, and Marine Biology, University of California Santa Barbara, Santa Barbara, CA 93106 USA
3. Department of Microbiology & Immunology, University of British Columbia, Vancouver, BC, V6T 1Z4, Canada
4. Hawkesbury Institute for the Environment, Western Sydney University, Richmond, NSW 2753, Australia

***Correspondence:** L. T. Stiemsma, 24255 Pacific Coast Hwy, Malibu CA, 90263, [leah.stiemsma@pepperdine.edu](mailto:leah.stiemsma@pepperdine.edu).

**Supplemental Methods and Results:**

**Methods:**

**Additional soil respiration details:** We used two systems of CO_2_ flux chambers so that all measurements could be completed within two to three hours, on the same sampling date, to improve comparisons between sites. We used three replicates of CO_2_ efflux at each collar to ensure consistency among measurements and check for proper alignment and chamber seal on collars. Each replicate measurement had a duration of 2.6 min including the dead band equilibration, thus each collar measurement required about 10 min to complete.

**Microbiome sequencing preprocessing:** Overlapping paired-end reads were processed into amplicon sequence variants (ASVs) with DADA2 and aligned to the SILVA reference database for taxonomy assignment [(1,2)](https://www.zotero.org/google-docs/?FdbCXp). Aside from adjusting the forward and reverse read trimming step (235 bp for forward reads and 200bp for reverse reads), we used the default DADA2 pipeline (raw ASVs can be found in **File S6**).

We used *decontam* in R to bioinformatically remove contaminate sequences from the soil samples [(3)](https://www.zotero.org/google-docs/?BKOg5S). This decontamination procedure removes contaminate reads based on their prevalence in the negative controls (n = 5). Bioinformatic decontamination reduced the dataset from 13,542 microbial taxa to 13,499 microbial taxa. We then selected ASVs classified as bacteria, reducing the dataset to 13,417 taxa. We then removed samples with fewer than 10,000 reads resulting in a dataset of 43 soil samples across the high and low burn sites in 2019, 2020, 2021, and 2023. We constructed a phylogenetic tree for this dataset using the *ape* package in R [(4)](https://www.zotero.org/google-docs/?Nsrqsw). Prior to analysis of relative abundance, we removed ASVs with less than 20 reads across all samples. We did not prune ASVs with less than 20 reads for analysis of alpha and beta diversity, but we did rarefy the dataset to the lowest sample’s read count of 15,019 reads.

**Results:**

**Comparison of high and low soil composition over time:** MaAsLin2 was also used to compare compositional diversity over time in the high and low burned soils (**File S3,** q < 0.25). For the years comparison, relative to 2019 there were no DA ASVs identified in either the high or low subsets. For the months comparison, relative to February 2019, the high subset resulted in 2202 DA ASVs, with many repeated taxa appearing throughout the month timepoints. The low subset resulted in 1209 DA ASVs. The increased number of DA taxa in high burned soils relative to low burned soils corroborates the more consistent taxonomic diversity observed in the low burned soils relative to high burned soils. These findings collectively support bacterial-type-conversion in the low burned soils.

**KOs in high and lowburn sites:** MaAsLin2 did not identify any DA KOs between high and low sites in 2019 (February or May) and only one KO was DA in 2020 (**File S4**). Supporting divergence of the high and low sites over time, MaAsLin2 identified 1,559 DA KOs in October 2021, and 575 DA KOs in 2023 (**File S4**).

**Comparison of high and low soil functional capacity over time:** We also used MaAsLin2 to identify DA KOs and pathways over time relative to 2019 (year only analysis, q < 0.25) and February 2019 (month and year analysis, q < 0.25). Comparing KOs across time points in the high burned soil relative to February 2019, MaAsLin2, identified 31 DA KOs with the majority of these DA KOs associated with the January 2023 timepoint (**File S4**). In the low burned soil, MaAsLin2 identified 9,728 DA KOs spread relatively evenly across all time points relative to February 2019 (**File S4**). Comparing KOs across years for high and low burn sites, MaAsLin2 identified 1,272 DA KOs in high soil and 1,816 DA KOs in low soil (**File S4**). MaAsLin2 identified 416 DA pathways for the month analysis in low burned soil and no DA pathways for the year analysis (**File S5**). In the high burned soil, for the analysis across years, MaAsLin2 identified three DA pathways (two in 2023 and one 2021, **File S5**). For the analysis across months in high burned soil, MaAsLin2 identified two DA pathways (both of a q value < 0.05, **File S5**). Of note, the mevalonate pathway was more abundant in 2023 and 2021 relative to 2019 for the analysis across years and in 2023 relative to February 2019 in the analysis across months (**File S5)**.

**References:**

[1. Callahan BJ, McMurdie PJ, Rosen MJ, Han AW, Johnson AJA, Holmes SP. DADA2: High-resolution sample inference from Illumina amplicon data. Nat Methods. 2016 Jul;13(7):581–3.](https://www.zotero.org/google-docs/?jh9dro)

[2. Quast C, Pruesse E, Yilmaz P, Gerken J, Schweer T, Yarza P, et al. The SILVA ribosomal RNA gene database project: improved data processing and web-based tools. Nucleic Acids Res. 2012 Nov 27;41(D1):D590–6.](https://www.zotero.org/google-docs/?jh9dro)

[3. Davis NM, Proctor DM, Holmes SP, Relman DA, Callahan BJ. Simple statistical identification and removal of contaminant sequences in marker-gene and metagenomics data. Microbiome. 2018 Dec;6(1):226.](https://www.zotero.org/google-docs/?jh9dro)

[4. Paradis E, Schliep K. ape 5.0: an environment for modern phylogenetics and evolutionary analyses in R. Schwartz R, editor. Bioinformatics. 2019 Feb 1;35(3):526–8.](https://www.zotero.org/google-docs/?jh9dro)

**Supplemental Tables:**

**Table S1: Number of soil samples collected in 2019, 2020, 2021, and 2023 in low and high severity burn sites for microbiome analysis**

| **Site** | **2019**  **(February & May)** | **November 2020** | **2021**  **(July and October)** | **January 2023** |
| --- | --- | --- | --- | --- |
| Low severity | 4 (2 February, 2 May) | 5 | 5 (3 July, 2 October) | 8 |
| High Severity | 4 (2 February, 2 May) | 4 | 5 (2 July, 3 October) | 6 |
| Total | 8 | 9 | 10 | 14 |

**Supplemental figures:**

**
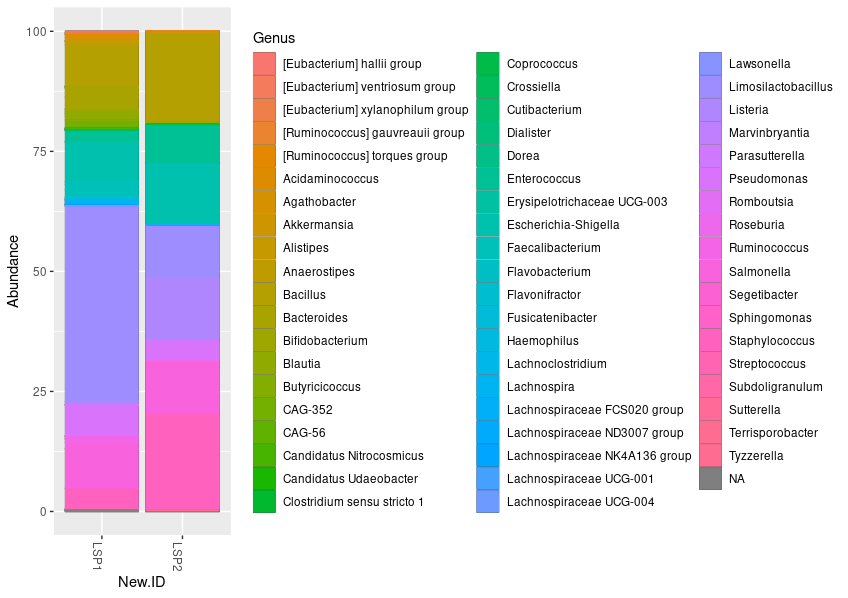
**

**Figure S1: Genus abundance in two positive control samples** (ZymoBIOMICS^TM^ microbial community standard (Catalog No. D6300)).


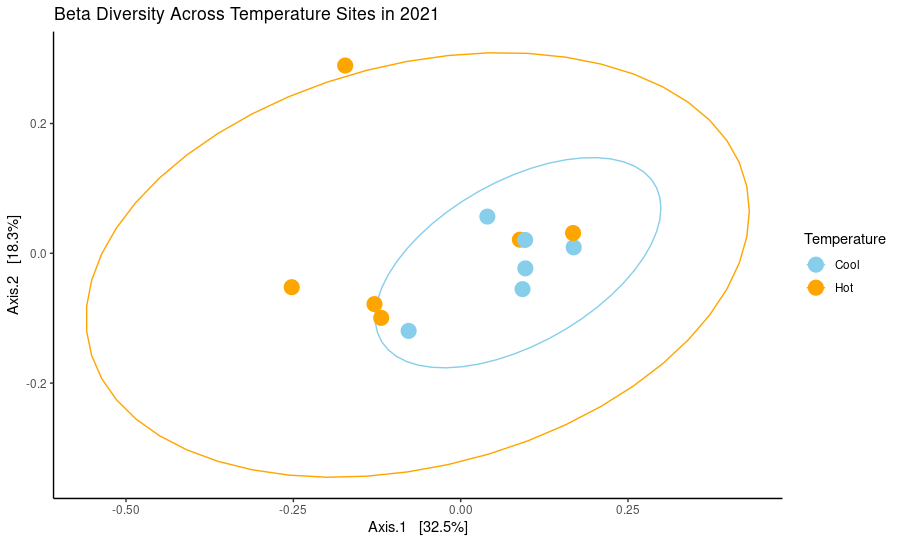

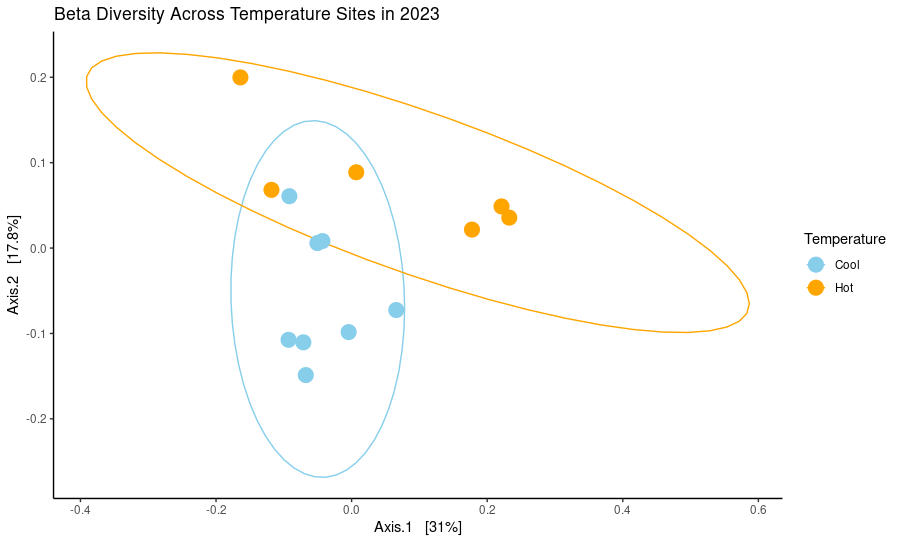

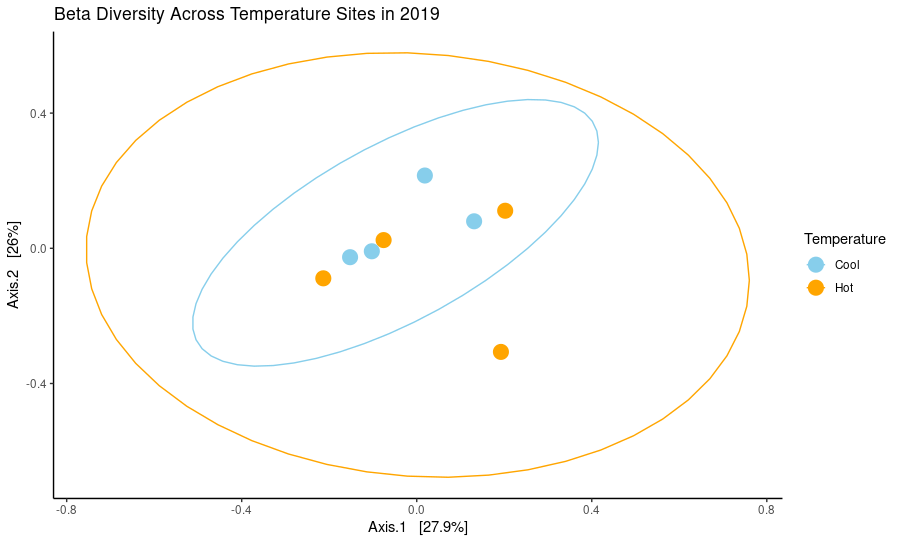

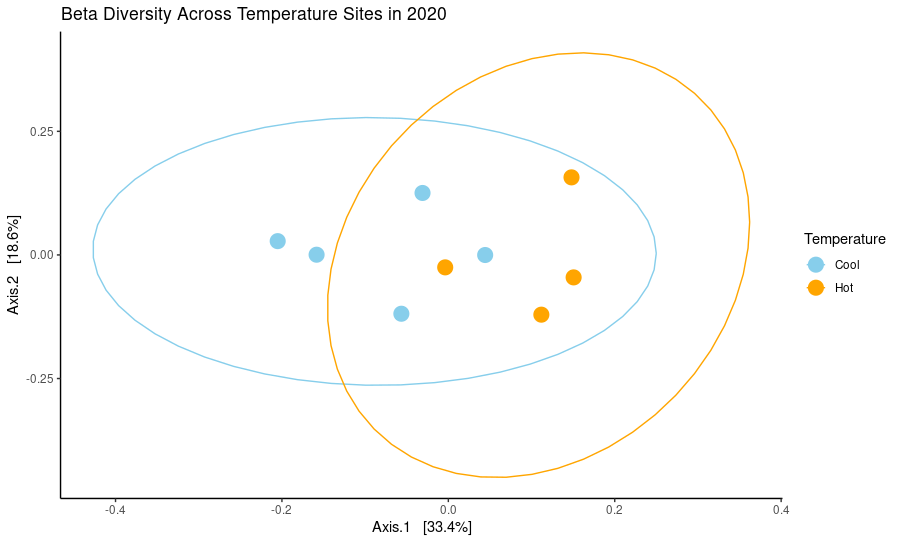


2019

2020

2021

2023

A

B

C

D

**Figure S2:** **Beta diversity based on weighted UniFrac distances** for A) 2019, B) 2020 (p<0.05), C) 2021, and D) 2023 (p < 0.05).

**Figure S3: Beta diversity across years in high and low severity burn sites.**
