# Supplementary material for: Effects of low wildfire burn severity due to pre-fire shrub thinning on the chaparral soil bacteriome in the Santa Monica Mountains of Southern California
Source: Microbiol Spectr. 2025 Jun 30;13(8):e00185-25. doi: 10.1128/spectrum.00185-25 (PMC12323652; doi:10.1128/spectrum.00185-25)
Supplement: File S10 — R Code. [file spectrum.00185-25-s0010.docx]

**File S10: R and terminal code for Macias et al.**

**#Preprocessing and decontamination - following DADA2 pipeline.**

#**Supp. Figure 1**, check the positive control

Pos <- subset_samples(Soil_2023, New.ID %in% c("LSP1", "LSP2") )

top100 <- names(sort(taxa_sums(Pos), decreasing=TRUE))[1:100]

top100_trs <- transform_sample_counts(Pos, function(OTU) OTU/sum(OTU))

top100_prune <- prune_taxa(top100, top100_trs)

merge = merge_samples(top100_prune, "New.ID")

sample_data(merge)$New.ID<- levels(sample_data(Pos)$New.ID)

merge.100 = transform_sample_counts(merge, function(x) 100 * x/sum(x))

p1 <- plot_bar(merge.100, "New.ID", "Abundance", "Genus")+ geom_bar(aes(color=Genus, fill=Genus), stat="identity", position='stack')

#Decontaminate data

nopos <- subset_samples(Soil_2023, New.ID != "LSP1") #subset out positive controls

nopos <- subset_samples(nopos, New.ID != "LSP2")

nopos

#phyloseq-class experiment-level object

#otu_table() OTU Table: [ 13542 taxa and 70 samples ]

#sample_data() Sample Data: [ 70 samples by 10 sample variables ]

#tax_table() Taxonomy Table: [ 13542 taxa by 6 taxonomic ranks ]

sample_data(nopos)$is.neg <- sample_data(nopos)$New.ID %in% c("LSC6", "LSH2O", "LSN1", "LSN2", "LSN3")

sample_data(nopos)

contamdf.prev <- isContaminant(nopos, method="prevalence", neg="is.neg")

#Warning message:

#In isContaminant(nopos, method = "prevalence", neg = "is.neg") :

#Removed 3 samples with zero total counts (or frequency).

table(contamdf.prev$contaminant)

#FALSE TRUE

#13522 20

head(which(contamdf.prev$contaminant))

#[1] 21 29 155 352 697 1106

contamdf.prev <- isContaminant(nopos, method="prevalence", neg="is.neg", threshold=0.5)

#Warning message:

#In isContaminant(nopos, method = "prevalence", neg = "is.neg", threshold = 0.5) :

#Removed 3 samples with zero total counts (or frequency).

table(contamdf.prev$contaminant)

FALSE TRUE

13499 43

decontam_soil <- prune_taxa(!contamdf.prev$contaminant, nopos)

decontam_soil

#phyloseq-class experiment-level object

#otu_table() OTU Table: [ 13499 taxa and 70 samples ]

#sample_data() Sample Data: [ 70 samples by 11 sample variables ]

#tax_table() Taxonomy Table: [ 13499 taxa by 6 taxonomic ranks ]

Soil_2023_onlyBacteria <- subset_taxa(decontam_soil, Kingdom == 'Bacteria') #keep only bacterial reads

#phyloseq-class experiment-level object

#otu_table() OTU Table: [ 13417 taxa and 70 samples ]

#sample_data() Sample Data: [ 70 samples by 11 sample variables ]

#tax_table() Taxonomy Table: [ 13417 taxa by 6 taxonomic ranks ]

#build a tree for the decontaminated dataset

random_tree = rtree(ntaxa(Soil_2023_onlyBacteria), rooted=TRUE, tip.label=taxa_names(Soil_2023))

#Warning message:

#In rtree(ntaxa(decontam_soil), rooted = TRUE, tip.label = taxa_names(Soil_2023)) :

#vector 'tip.label' longer than 'n': was shorten

Soil2023_treed = merge_phyloseq(Soil_2023_onlyBacteria, random_tree)

Soil2023_treed

#phyloseq-class experiment-level object

#otu_table() OTU Table: [ 13293 taxa and 70 samples ]

#sample_data() Sample Data: [ 70 samples by 11 sample variables ]

#tax_table() Taxonomy Table: [ 13293 taxa by 6 taxonomic ranks ]

#phy_tree() Phylogenetic Tree: [ 13293 tips and 13292 internal nodes

#Remove samples with less than 10,000 reads

sort(phyloseq::sample_sums(Soil2023_treed)) #sort by read counts

#LS27 LSC6 LSH2O LSN1 LSN3 LSN2 LS07 LS31 LS10 LS20 LS25 LS08 LS13 LS02 LS06 LS17 LS28 LS18 LS44

#0 0 0 1 207 234 2675 6060 15019 15143 21596 29289 29707 30888 31158 34562 34823 37060 37351

#LS03 LS12 LS29 LS41 LS47 LS42 LS01 LS14 LS68 LS37 LS50 LS51 LS11 LS09 LS15 LS56 LS16 LS63 LS53

#37612 38325 39185 39234 40494 40509 40824 41339 42113 42224 42390 42449 42654 42772 42933 43424 44360 44658 44986

#LS21 LS52 LS40 LS39 LS23 LS62 LS57 LS36 LS65 LS43 LS19 LS30 LS54 LS32 LS33 LS60 LS49 LS34 LS22

#45315 45793 45859 46236 46828 46830 46964 47204 47295 47471 47516 48121 48690 49547 49735 51436 52325 53033 53464

#LS24 LS46 LS59 LS35 LS05 LS04 LS48 LS38 LS64 LS67 LS58 LS45 LS26

#53874 54036 54176 54392 55331 55400 56309 56514 58999 59491 63147 63663 105636

Soil2023_treed_pruned <- subset_samples(Soil2023_treed, sample_sums(Soil2023_treed) > 10000)

Soil2023_treed_pruned #dataset for relative abundance/maaslin/lefse analysis

phyloseq-class experiment-level object

#otu_table() OTU Table: [ 13293 taxa and 62 samples ]

#sample_data() Sample Data: [ 62 samples by 11 sample variables ]

#tax_table() Taxonomy Table: [ 13293 taxa by 6 taxonomic ranks ]

#phy_tree() Phylogenetic Tree: [ 13293 tips and 13292 internal nodes ]

soil_relativeabund_pruned <- prune_taxa(taxa_sums(soil)>=20, soil)

soil_relativeabund_pruned

#otu_table() OTU Table: [ 7639 taxa and 62 samples ]

#sample_data() Sample Data: [ 62 samples by 11 sample variables ]

#tax_table() Taxonomy Table: [ 7639 taxa by 6 taxonomic ranks ]

#phy_tree() Phylogenetic Tree: [ 7639 tips and 7638 internal nodes ]

#Create rarefied dataset for alpha/beta diversity

saveRDS(Soil2023_treed_pruned, "Soil2023_forrelativeabund.RDS") #relative abundance dataset

Soil_2023_treed_pruned_rarefied = rarefy_even_depth(Soil2023_treed_pruned, rngseed=1, sample.size = 15019, replace = FALSE, verbose = TRUE) #dataset used to rarefy is not pruned for ASVs, but is pruned for samples with less than 10,000 reads. Used for alpha and beta diversity

sort(phyloseq::sample_sums(Soil_2023_treed_pruned_rarefied)) #sort by read counts

#LS01 LS02 LS03 LS04 LS05 LS06 LS08 LS09 LS10 LS11 LS12 LS13 LS14 LS15 LS16 LS17 LS18 LS19 LS20 LS21 LS22 LS23

#15019 15019 15019 15019 15019 15019 15019 15019 15019 15019 15019 15019 15019 15019 15019 15019 15019 15019 15019 15019 15019 15019

#LS24 LS25 LS26 LS28 LS29 LS30 LS32 LS33 LS34 LS35 LS36 LS37 LS38 LS39 LS40 LS41 LS42 LS43 LS44 LS45 LS46 LS47

#15019 15019 15019 15019 15019 15019 15019 15019 15019 15019 15019 15019 15019 15019 15019 15019 15019 15019 15019 15019 15019 15019

#LS48 LS49 LS50 LS51 LS52 LS53 LS54 LS56 LS57 LS58 LS59 LS60 LS62 LS63 LS64 LS65 LS67 LS68

#15019 15019 15019 15019 15019 15019 15019 15019 15019 15019 15019 15019 15019 15019 15019 15019 15019 15019

saveRDS(Soil_2023_treed_pruned_rarefied, "Soil2023_alphabeta.RDS") #alpha and beta div dataset

**#Figure 1, File S1**

**#Soil respiration**

soil_resp <- read.csv("resp_20192021.csv", header = T)

soil_resp$burn_time <- paste(soil_resp$date2, soil_resp$site) #create combined variable

shapiro.test(soil_resp$Flux_to) #normality testing

kw <- kruskal.test(soil_resp$Flux_to ~ soil_resp$burn_time, data = soil_resp)

kw

dunnTest(soil_resp$Flux_to ~ soil_resp$burn_time,

data=soil_resp,

method="bonferroni")

#plot

p<-ggplot(data=soil_resp, aes(x=date2, y=Flux_to, fill=site)) +

geom_boxplot() + scale_fill_manual(values=c("skyblue",

)) + theme_classic() + facet_wrap(~site) + theme_classic() + xlab("Date") + ylab("CO2 Flux (µmol m-2 s-1) ")+scale_x_discrete(labels = c("May, 2019", "June 4, 2019", "June 11, 2019", "June 17, 2019", "June 28, 2019", "July, 2019", "Dec 12, 2020", "Feb 27, 2021", "March 19, 2021")) + coord_cartesian(ylim=c(0,8)) + geom_jitter(color="black", size=1) + scale_y_continuous(breaks = seq(0,8, by=0.5)) + theme(legend.text=element_text(size=12), axis.text=element_text(size=16), axis.title=element_text(size=18), strip.text = element_text(size=25)) #repeat for year, replace with x=year

**#Figure 2, File S1**

**#Nutrients and pH**

soilnutr_combined <- read.csv("soil_nutrients_20192021_combined.csv", header = T)

soilnutr_combined$high_year <- paste(soilnutr_combined$burn, soilnutr_combined$Year) #create combined variable

shapiro.test(soilnutr_combined$OrgMatt) #substitute variable

kw <- kruskal.test(soilnutr_combined$OrgMatt ~ soilnutr_combined$high_year, data = soilnutr_combined) #non parametric

dunnTest(soilnutr_combined$K ~ soilnutr_combined$high_year,

data=soilnutr_combined,

method="bonferroni") #non parametric

aov <- aov(soilnutr_combined$pH ~ soilnutr_combined$high_year, data = soilnutr_combined) #parametric

TukeyHSD(aov) #parametric

**#Figure 3 (E-H), Figure S2, File S1**

totalsoil <- readRDS("Soil2023_alphabeta.RDS")

sample_data(totalsoil)

irrigation_subset <- subset_samples(totalsoil, Irrigation %in% c("No"))

sample_data(irrigation_subset)

burn_subset <- subset_samples(irrigation_subset, Burn %in% c("Burnd", "Burned"))

sample_data(burn_subset)

temp_subset <- subset_samples(burn_subset, Temperature %in% c("high","low"))

sample_data(temp_subset)

temp_subset$high_year <- paste(temp_subset$burn, temp_subset$Year) #create combined variable

shapiro.test(temp_subset$Shannon) #substitute diversity variable (Shannon or Chao1)

kw <- kruskal.test(temp_subset$Shannon ~ temp_subset$high_year, data = temp_subset) #non parametric

dunnTest(temp_subset$Shanon ~ temp_subset$high_year,

data=temp_subset,

method="bonferroni") #non parametric

aov <- aov(temp_subset$Chao1 ~ temp_subset$high_year, data = soilnutr_combined) #parametric

TukeyHSD(aov) #parametric

**#Beta diversity plots (Figure 3, A-D)**

**#2019**

y_2019 <- subset_samples(temp_subset, as.factor(Year) %in% c("2019"))

sample_data(y_2019)

ord_2019 <- ordinate(y_2019, "PCoA", "Unifrac")

plot_2019_ord <- plot_ordination(temp_subset, ord_2019, type="samples", color="Temperature", title="Beta Diversity Across Temperature Sites in 2019") + stat_ellipse(level= 0.95) + geom_point(size=5) + theme_classic()

plot_2019_ord + scale_color_manual(values = palette)

**#2020**

y_2020 <- subset_samples(temp_subset, as.factor(Year) %in% c("2020"))

sample_data(y_2020)

ord_2020 <- ordinate(y_2020, "PCoA", "Unifrac")

plot_2020_ord <- plot_ordination(temp_subset, ord_2020, type="samples", color="Temperature", title="Beta Diversity Across Temperature Sites in 2020") + stat_ellipse(level= 0.95) + geom_point(size=5) + theme_classic()

plot_2020_ord + scale_color_manual(values = palette)

**#2021**

y_2021 <- subset_samples(temp_subset, as.factor(Year) %in% c("2021"))

sample_data(y_2021)

ord_2021 <- ordinate(y_2021, "PCoA", "Unifrac")

plot_2021_ord <- plot_ordination(temp_subset, ord_2021, type="samples", color="Temperature", title="Beta Diversity Across Temperature Sites in 2021") + stat_ellipse(level= 0.95) + geom_point(size=5) + theme_classic()

plot_2021_ord + scale_color_manual(values = palette)

**#2023**

y_2023 <- subset_samples(temp_subset, as.factor(Year) %in% c("2023"))

sample_data(y_2023)

ord_2023 <- ordinate(y_2023, "PCoA", "Unifrac")

plot_2023_ord <- plot_ordination(temp_subset, ord_2023, type="samples", color="Temperature", title="Beta Diversity Across Temperature Sites in 2023") + stat_ellipse(level= 0.95) + geom_point(size=5) + theme_classic()

plot_2023_ord + scale_color_manual(values = palette)

**#beta diversity stats**

#Year subsets

y2019 <- subset_samples(soil_div, Year%in%c("2019")) #w betadisper nonsig

y2020 <- subset_samples(soil_div, Year%in%c("2020"))#w and unweighted betadispir nonsig

y2021<- subset_samples(soil_div, Year%in%c("2021")) #w and unweighted betadispir nonsig

y2023<- subset_samples(soil_div, Year%in%c("2023")) #both nonsig betadisper

#replace with each subset for stats on beta div. Run betadispr first, only run adonis if betadispr >0.05.

low_div_year <- subset_samples(soil_div, Temperature%in%c("low")) #do for high as well

distance_2019 = distance(y2019, method = "wunifrac") #change to unifrac for unweighted.

groups <- sample_data(distance_2019)$Temperature

mod <- betadisper(distance_2019, groups)

permutest(mod)

mod.HSD <- TukeyHSD(mod)

plot(mod.HSD) #replace wunifrac with unifrac or add weighted = FALSE

adonis2 <- adonis2(distance_2019 ~ Temperature,as(sample_data(distance_2019), "data.frame"))

adonis2

For stats across years…

low_div_year <- subset_samples(soil_div, Temperature%in%c("low")) #do for high as well

distance= distance(low_div_year, method = "wunifrac") #unweighted as well

groups <- sample_data(low_div_year)$Year

mod <- betadisper(distance, groups)

permutest(mod)

mod.HSD <- TukeyHSD(mod)

plot(mod.HSD) #replace wunifrac with unifrac or add weighted = FALSE

adonis2 <- adonis2(distance~ Year,as(sample_data(low_div_year), "data.frame"))

adonis2

**#Figure 4**

**#relative abundance years + month/year (across within temp subsets)**

irrigation_subset_RA <- subset_samples(totalsoilRA, Irrigation %in% c("No")) #applicable if using the raw fastqs (these samples are not included in the ASV, KO, or pathway supplemental files.

sample_data(irrigation_subset_RA)

burn_subset_RA <- subset_samples(irrigation_subset_RA, Burn %in% c("Burnd", "Burned"))

sample_data(burn_subset_RA)

temp_subset_RA <- subset_samples(burn_subset_RA, Temperature %in% c("high","low"))

sample_data(temp_subset_RA)

sample_data(temp_subset_RA)$Year <- as.factor(sample_data(temp_subset_RA)$Year)#make Year a factor

**#years high and low relative abundance**

top100 <- names(sort(taxa_sums(temp_subset_RA), decreasing=TRUE))[1:100]

top100_trs <- transform_sample_counts(temp_subset_RA, function(OTU) OTU/sum(OTU))

top100_prune <- prune_taxa(top100, top100_trs)

high <- subset_samples(top100_prune, Temperature %in% c("high"))

merge = merge_samples(high, "Year")

sample_data(merge)$Year<- levels(sample_data(temp_subset_RA)$Year)

merge.100 = transform_sample_counts(merge, function(x) 100 * x/sum(x))

pP_high_years <- plot_bar(merge.100, "Year", "Abundance", "Phylum") + geom_bar(aes(color=Phylum, fill=Phylum), stat="identity", position='stack') + theme_classic()

pP_high_years

low <- subset_samples(top100_prune, Temperature %in% c("low"))

merge = merge_samples(low, "Year")

sample_data(merge)$Year<- levels(sample_data(temp_subset_RA)$Year)

merge.100 = transform_sample_counts(merge, function(x) 100 * x/sum(x))

pP_low_years <- plot_bar(merge.100, "Year", "Abundance", "Phylum") + geom_bar(aes(color=Phylum, fill=Phylum), stat="identity", position='stack') + theme_classic()

pP_low_years

**#month/year high and low relative abundance**

monthyear_RA <- subset_samples(temp_subset_RA, month_year2 %in% c("Feb_2019", "May_2019", "2020", "July_2021", "Oct_2021", "Jan_2023"))

top100 <- names(sort(taxa_sums(monthyear_RA), decreasing=TRUE))[1:100]

top100_trs <- transform_sample_counts(monthyear_RA, function(OTU) OTU/sum(OTU))

top100_prune <- prune_taxa(top100, top100_trs)

high <- subset_samples(top100_prune, Temperature %in% c("high"))

merge = merge_samples(high, "month_year2")

sample_data(merge)$month_year2<- levels(sample_data(monthyear_RA)$month_year2)

sample_data(merge)$month_year2 <- factor(sample_data(merge)$month_year2,

levels = c("Feb_2019", "May_2019", "2020", "July_2021", "Oct_2021", "Jan_2023"))

merge.100 = transform_sample_counts(merge, function(x) 100 * x/sum(x))

pP_high_monthyear <- plot_bar(merge.100, "month_year2", "Abundance", "Phylum") + geom_bar(aes(color=Phylum, fill=Phylum), stat="identity", position='stack') + theme_classic()

pP_high_monthyear + scale_x_discrete(labels = c("February 2019", "May 2019", "2020", "July 2021", "October 2021", "January 2023")) + xlab("Month and Year")

low <- subset_samples(top100_prune, Temperature %in% c("low"))

merge = merge_samples(low, "month_year2")

sample_data(merge)$month_year2<- levels(sample_data(monthyear_RA)$month_year2)

sample_data(merge)$month_year2 <- factor(sample_data(merge)$month_year2,

levels = c("Feb_2019", "May_2019", "2020", "July_2021", "Oct_2021", "Jan_2023"))

merge.100 = transform_sample_counts(merge, function(x) 100 * x/sum(x))

pP_low_monthyear <- plot_bar(merge.100, "month_year2", "Abundance", "Phylum") + geom_bar(aes(color=Phylum, fill=Phylum), stat="identity", position='stack') + theme_classic()

pP_low_monthyear + theme(axis.text.x = element_text(angle = 30, vjust = 1, hjust=1)) + scale_x_discrete(labels = c("February 2019", "May 2019", "2020", "July 2021", "October 2021", "January 2023")) + xlab("Month and Year")

**#Maaslin stats subset**

irrigation_maaslin <- subset_samples(maaslin_rerun, Irrigation %in% c("No")) #applicable if using the raw fastqs (these samples are not included in the ASV, KO, or pathway supplemental files.

sample_data(irrigation_maaslin)

burn_maaslin <- subset_samples(irrigation_maaslin, Burn %in% c("Burnd", "Burned"))

sample_data(burn_maaslin)

temp_maaslin <- subset_samples(burn_maaslin, Temperature %in% c("high","low"))

sample_data(temp_maaslin)

sample_data(temp_maaslin)$Year <- as.factor(sample_data(temp_maaslin)$Year) #make Year a factor

str(sample_data(temp_maaslin))

**#Maaslin Stats and graphs years + month/year, Figure 5**

#2019

maaslin_2019 <- subset_samples(temp_maaslin, Year %in% c("2019"))

sample_data(maaslin_2019)

write.csv(maaslin_2019@tax_table, 'maaslin_2019_ASVs.csv')

subset_RA_2019_taxtable <- read.csv('maaslin_2019_ASVs.csv', header=TRUE)

ID <- (1:7639)

subset_RA_2019_taxtable$ID<- ID

View(subset_RA_2019_taxtable)

analysisdata_pruned_abund_2019 <- microbiome::transform(maaslin_2019,

transform = "compositional",

target = "OTU", shift = 0,

scale = 1) #transform ASVs to relative abundance from your phyloseq subset

input_data_2019 <- as.data.frame(analysisdata_pruned_abund_2019@otu_table) #extract ASV table from that new transformed dataset

input_data_2019 <- as.data.frame(t(input_data_2019)) #transpose to correct orientation

rownames(input_data_2019) <- as.factor(subset_RA_2019_taxtable$ID)

meta_2019 <- as.matrix(analysisdata_pruned_abund_2019@sam_data) #extract metadata

meta_2019 <- as.data.frame(meta_2019) #make metadata a dataframe

fit_data_2019 = Maaslin2(input_data = input_data_2019, input_metadata = meta_2019, output = "maaslin2_output_2019", fixed_effects = c("Temperature"),plot_heatmap = TRUE) #run maaslin

fit_data_df_2019 <- as.data.frame(fit_data_2019$results) #pull out results only from maaslin output

fit_data_df_sig_2019 <- subset(fit_data_df_2019, qval <= 0.25) #pull out only significant results using q <= 0.25

maaslin2_2019 <- ggplot(fit_data_df_sig_2019, aes(x=coef, y=feature, color = value, shape = value)) +

theme_classic() +xlab("Coefficient") +

geom_errorbar(aes(xmin=coef-stderr,xmax=coef+stderr), width=.2,position=position_dodge(0.25)) +

geom_point(size=3, position=position_dodge(0.25),aes(fill=value, color=value))+

ylab("") + ggtitle("")+

xlab("") + xlab("Coefficient") + ylab("Feature") + scale_fill_manual(values=c('orange'))+ scale_color_manual(values=c('orange')) +

scale_shape_manual(values=seq(0,10))+

theme(axis.title.x=element_text(size=10)) +

theme(axis.title.y=element_text(size=10)) +

theme(axis.text.x=element_text(size=10)) +

theme(axis.text.y=element_text(size=8)) +theme(legend.text=element_text(size=10)) +

theme(legend.title=element_text(size=10))

maaslin2_2019 #view plot

#2020

maaslin_2020 <- subset_samples(temp_maaslin, Year %in% c("2020"))

sample_data(maaslin_2020)

write.csv(maaslin_2020@tax_table, 'maaslin_2020_ASVs.csv')

subset_RA_2020_taxtable <- read.csv('maaslin_2020_ASVs.csv', header=TRUE)

ID <- (1:7639)

subset_RA_2020_taxtable$ID<- ID

View(subset_RA_2020_taxtable)

analysisdata_pruned_abund_2020 <- microbiome::transform(maaslin_2020,

transform = "compositional",

target = "OTU", shift = 0,

scale = 1) #transform ASVs to relative abundance from your phyloseq subset

input_data_2020 <- as.data.frame(analysisdata_pruned_abund_2020@otu_table) #extract ASV table from that new transformed dataset

input_data_2020 <- as.data.frame(t(input_data_2020)) #transpose to correct orientation

rownames(input_data_2020) <- as.factor(subset_RA_2020_taxtable$ID)#replace ASV nucleotides with ID column from your taxa table (edited with above code)

meta_2020 <- as.matrix(analysisdata_pruned_abund_2020@sam_data) #extract metadata

meta_2020 <- as.data.frame(meta_2020) #make metadata a dataframe

fit_data_2020 = Maaslin2(input_data = input_data_2020, input_metadata = meta_2020, output = "maaslin2_output_2020", fixed_effects = c("Temperature"),plot_heatmap = TRUE) #run maaslin

fit_data_df_2020 <- as.data.frame(fit_data_2020$results)#pull out results only from maaslin output

fit_data_df_sig_2020 <- subset(fit_data_df_2020, qval <= 0.25) #pull out only significant results using q <= 0.25

maaslin2_2020 <- ggplot(fit_data_df_sig_2020, aes(x=coef, y=feature, color = value, shape = value)) +

theme_classic() +xlab("Coefficient") +

geom_errorbar(aes(xmin=coef-stderr,xmax=coef+stderr), width=.2,position=position_dodge(0.25)) +

geom_point(size=3, position=position_dodge(0.25),aes(fill=value, color=value))+

ylab("") + ggtitle("")+

xlab("") + xlab("Coefficient") + ylab("Feature") + scale_fill_manual(values=c('orange'))+ scale_color_manual(values=c('orange')) +

scale_shape_manual(values=seq(0,10))+

theme(axis.title.x=element_text(size=10)) +

theme(axis.title.y=element_text(size=10)) +

theme(axis.text.x=element_text(size=10)) +

theme(axis.text.y=element_text(size=8)) +theme(legend.text=element_text(size=10)) +

theme(legend.title=element_text(size=10))

maaslin2_2020 #view plot

#2021

maaslin_2021 <- subset_samples(temp_maaslin, Year %in% c("2021"))

sample_data(maaslin_2021)

write.csv(maaslin_2021@tax_table, 'maaslin_2021_ASVs.csv')

subset_RA_2021_taxtable <- read.csv('maaslin_2021_ASVs.csv', header=TRUE)

ID <- (1:7639)

subset_RA_2021_taxtable$ID<- ID

View(subset_RA_2021_taxtable)

analysisdata_pruned_abund_2021 <- microbiome::transform(maaslin_2021,

transform = "compositional",

target = "OTU", shift = 0,

scale = 1) #transform ASVs to relative abundance from your phyloseq subset

input_data_2021 <- as.data.frame(analysisdata_pruned_abund_2021@otu_table) #extract ASV table from that new transformed dataset

input_data_2021 <- as.data.frame(t(input_data_2021)) #transpose to correct orientation

rownames(input_data_2021) <- as.factor(subset_RA_2021_taxtable$ID)

meta_2021 <- as.matrix(analysisdata_pruned_abund_2021@sam_data) #extract metadata

meta_2021 <- as.data.frame(meta_2021) #make metadata a dataframe

fit_data_2021 = Maaslin2(input_data = input_data_2021, input_metadata = meta_2021, output = "maaslin2_output_2021", fixed_effects = c("Temperature"),plot_heatmap = TRUE) #run maaslin

fit_data_df_2021 <- as.data.frame(fit_data_2021$results) #pull out results only from maaslin output

fit_data_df_sig_2021 <- subset(fit_data_df_2021, qval <= 0.25) #pull out only significant results using q <= 0.25

maaslin2_2021 <- ggplot(fit_data_df_sig_2021, aes(x=coef, y=feature, color = value, shape = value)) +

theme_classic() +xlab("Coefficient") +

geom_errorbar(aes(xmin=coef-stderr,xmax=coef+stderr), width=.2,position=position_dodge(0.25)) +

geom_point(size=3, position=position_dodge(0.25),aes(fill=value, color=value))+

ylab("") + ggtitle("")+

xlab("") + xlab("Coefficient") + ylab("Feature") + scale_fill_manual(values=c('orange'))+ scale_color_manual(values=c('orange')) +

scale_shape_manual(values=seq(0,10))+

theme(axis.title.x=element_text(size=10)) +

theme(axis.title.y=element_text(size=10)) +

theme(axis.text.x=element_text(size=10)) +

theme(axis.text.y=element_text(size=8)) +theme(legend.text=element_text(size=10)) +

theme(legend.title=element_text(size=10))

maaslin2_2021 #view plot

#2023

maaslin_2023 <- subset_samples(temp_maaslin, Year %in% c("2023"))

sample_data(maaslin_2023)

write.csv(maaslin_2023@tax_table, 'maaslin_2023_ASVs.csv')

subset_RA_2023_taxtable <- read.csv('maaslin_2023_ASVs.csv', header=TRUE)

ID <- (1:7639)

subset_RA_2023_taxtable$ID<- ID

View(subset_RA_2023_taxtable)

analysisdata_pruned_abund_2023 <- microbiome::transform(maaslin_2023,

transform = "compositional",

target = "OTU", shift = 0,

scale = 1)

input_data_2023 <- as.data.frame(analysisdata_pruned_abund_2023@otu_table) #extract ASV table from that new transformed dataset

input_data_2023 <- as.data.frame(t(input_data_2023)) #transpose to correct orientation

rownames(input_data_2023) <- as.factor(subset_RA_2023_taxtable$ID)

meta_2023 <- as.matrix(analysisdata_pruned_abund_2023@sam_data) #extract metadata

meta_2023 <- as.data.frame(meta_2023) #make metadata a dataframe

fit_data_2023 = Maaslin2(input_data = input_data_2023, input_metadata = meta_2023, output = "maaslin2_output_2023", fixed_effects = c("Temperature"),plot_heatmap = TRUE) #run maaslin

fit_data_df_2023 <- as.data.frame(fit_data_2023$results) #pull out results only from maaslin output

fit_data_df_sig_2023 <- subset(fit_data_df_2023, qval <= 0.25) #pull out only significant results using q <= 0.25

maaslin2_2023 <- ggplot(fit_data_df_sig_2023, aes(x=coef, y=feature, color = value, shape = value)) +

theme_classic() +xlab("Coefficient") +

geom_errorbar(aes(xmin=coef-stderr,xmax=coef+stderr), width=.2,position=position_dodge(0.25)) +

geom_point(size=3, position=position_dodge(0.25),aes(fill=value, color=value))+

ylab("") + ggtitle("")+

xlab("") + xlab("Coefficient") + ylab("Feature") + scale_fill_manual(values=c('orange'))+ scale_color_manual(values=c('orange')) +

scale_shape_manual(values=seq(0,10))+

theme(axis.title.x=element_text(size=10)) +

theme(axis.title.y=element_text(size=10)) +

theme(axis.text.x=element_text(size=10)) +

theme(axis.text.y=element_text(size=8)) +theme(legend.text=element_text(size=10)) +

theme(legend.title=element_text(size=10))

maaslin2_2023 #view plot

#february 2019

february2019 <- subset_samples(maaslin_2019, Month_Year %in% c("Feb_2019"))

sample_data(february2019)

write.csv(february2019@tax_table, 'february2019_ASVs.csv')

feb2019_taxtable <- read.csv('february2019_ASVs.csv', header=TRUE)

ID <- (1:7639)

feb2019_taxtable$ID<- ID

View(feb2019_taxtable)

analysisdata_pruned_abund_feb19 <- microbiome::transform(february2019,

transform = "compositional",

target = "OTU", shift = 0,

scale = 1)

input_data_feb19 <- as.data.frame(analysisdata_pruned_abund_feb19@otu_table) #extract ASV table from that new transformed dataset

input_data_feb19 <- as.data.frame(t(input_data_feb19)) #transpose to correct orientation

rownames(input_data_feb19) <- as.factor(feb2019_taxtable$ID)

meta_feb19 <- as.matrix(analysisdata_pruned_abund_feb19@sam_data) #extract metadata

meta_feb19 <- as.data.frame(meta_feb19) #make metadata a dataframe

fit_data_feb19 = Maaslin2(input_data = input_data_feb19, input_metadata = meta_feb19, output = "maaslin2_output_feb19", fixed_effects = c("Temperature"),plot_heatmap = TRUE) #run maaslin

fit_data_df_feb19 <- as.data.frame(fit_data_feb19$results) #pull out results only from maaslin output

fit_data_df_sig_feb19 <- subset(fit_data_df_feb19, qval <= 0.25) #pull out only significant results using q <= 0.25

maaslin2_feb19 <- ggplot(fit_data_df_sig_feb19, aes(x=coef, y=feature, color = value, shape = value)) +

theme_classic() +xlab("Coefficient") +

geom_errorbar(aes(xmin=coef-stderr,xmax=coef+stderr), width=.2,position=position_dodge(0.25)) +

geom_point(size=3, position=position_dodge(0.25),aes(fill=value, color=value))+

ylab("") + ggtitle("")+

xlab("") + xlab("Coefficient") + ylab("Feature") + scale_fill_manual(values=c('orange'))+ scale_color_manual(values=c('orange')) +

scale_shape_manual(values=seq(0,10))+

theme(axis.title.x=element_text(size=10)) +

theme(axis.title.y=element_text(size=10)) +

theme(axis.text.x=element_text(size=10)) +

theme(axis.text.y=element_text(size=8)) +theme(legend.text=element_text(size=10)) +

theme(legend.title=element_text(size=10))

maaslin2_feb19 #view plot

#may 2019

may2019 <- subset_samples(maaslin_2019, Month_Year %in% c("May_2019"))

sample_data(may2019)

write.csv(may2019@tax_table, 'may2019_ASVs.csv')

may2019_taxtable <- read.csv('may2019_ASVs.csv', header=TRUE)

ID <- (1:7639)

may2019_taxtable$ID<- ID

View(may2019_taxtable)

analysisdata_pruned_abund_may19 <- microbiome::transform(may2019,

transform = "compositional",

target = "OTU", shift = 0,

scale = 1)

input_data_may19 <- as.data.frame(analysisdata_pruned_abund_may19@otu_table) #extract ASV table from that new transformed dataset

input_data_may19 <- as.data.frame(t(input_data_may19)) #transpose to correct orientation

rownames(input_data_may19) <- as.factor(may2019_taxtable$ID)

meta_may19 <- as.matrix(analysisdata_pruned_abund_may19@sam_data) #extract metadata

meta_may19 <- as.data.frame(meta_may19) #make metadata a dataframe

fit_data_may19 = Maaslin2(input_data = input_data_may19, input_metadata = meta_may19, output = "maaslin2_output_may19", fixed_effects = c("Temperature"),plot_heatmap = TRUE) #run maaslin

fit_data_df_may19 <- as.data.frame(fit_data_may19$results) #pull out results only from maaslin output

fit_data_df_sig_may19 <- subset(fit_data_df_may19, qval <= 0.25) #pull out only significant results using q <= 0.25

maaslin2_may19 <- ggplot(fit_data_df_sig_may19, aes(x=coef, y=feature, color = value, shape = value)) +

theme_classic() +xlab("Coefficient") +

geom_errorbar(aes(xmin=coef-stderr,xmax=coef+stderr), width=.2,position=position_dodge(0.25)) +

geom_point(size=3, position=position_dodge(0.25),aes(fill=value, color=value))+

ylab("") + ggtitle("")+

xlab("") + xlab("Coefficient") + ylab("Feature") + scale_fill_manual(values=c('orange'))+ scale_color_manual(values=c('orange')) +

scale_shape_manual(values=seq(0,10))+

theme(axis.title.x=element_text(size=10)) +

theme(axis.title.y=element_text(size=10)) +

theme(axis.text.x=element_text(size=10)) +

theme(axis.text.y=element_text(size=8)) +theme(legend.text=element_text(size=10)) +

theme(legend.title=element_text(size=10))

maaslin2_may19 #view plot

#july2021

july2021 <- subset_samples(maaslin_2021, Month_Year %in% c("July_2021"))

sample_data(july2021)

write.csv(july2021@tax_table, 'july2021_ASVs.csv')

july2021_taxtable <- read.csv('july2021_ASVs.csv', header=TRUE)

ID <- (1:7639)

july2021_taxtable$ID<- ID

View(july2021_taxtable)

analysisdata_pruned_abund_july21 <- microbiome::transform(july2021,

transform = "compositional",

target = "OTU", shift = 0,

scale = 1)

input_data_july21 <- as.data.frame(analysisdata_pruned_abund_july21@otu_table) #extract ASV table from that new transformed dataset

input_data_july21 <- as.data.frame(t(input_data_july21)) #transpose to correct orientation

rownames(input_data_july21) <- as.factor(july2021_taxtable$ID)

meta_july21 <- as.matrix(analysisdata_pruned_abund_july21@sam_data) #extract metadata

meta_july21 <- as.data.frame(meta_july21) #make metadata a dataframe

fit_data_july21 = Maaslin2(input_data = input_data_july21, input_metadata = meta_july21, output = "maaslin2_output_july21", fixed_effects = c("Temperature"),plot_heatmap = TRUE) #run maaslin

fit_data_df_july21 <- as.data.frame(fit_data_july21$results) #pull out results only from maaslin output

fit_data_df_sig_july21 <- subset(fit_data_df_july21, qval <= 0.25) #pull out only significant results using q <= 0.25

maaslin2_july21 <- ggplot(fit_data_df_sig_july21, aes(x=coef, y=feature, color = value, shape = value)) +

theme_classic() +xlab("Coefficient") +

geom_errorbar(aes(xmin=coef-stderr,xmax=coef+stderr), width=.2,position=position_dodge(0.25)) +

geom_point(size=3, position=position_dodge(0.25),aes(fill=value, color=value))+

ylab("") + ggtitle("")+

xlab("") + xlab("Coefficient") + ylab("Feature") + scale_fill_manual(values=c('orange'))+ scale_color_manual(values=c('orange')) +

scale_shape_manual(values=seq(0,10))+

theme(axis.title.x=element_text(size=10)) +

theme(axis.title.y=element_text(size=10)) +

theme(axis.text.x=element_text(size=10)) +

theme(axis.text.y=element_text(size=8)) +theme(legend.text=element_text(size=10)) +

theme(legend.title=element_text(size=10))

maaslin2_july21 #view plot

#october2021

october2021 <- subset_samples(maaslin_2021, Month_Year %in% c("Oct_2021"))

sample_data(october2021)

write.csv(october2021@tax_table, 'oct2021_ASVs.csv')

oct2021_taxtable <- read.csv('oct2021_ASVs.csv', header=TRUE)

ID <- (1:7639)

oct2021_taxtable$ID<- ID

View(oct2021_taxtable)

analysisdata_pruned_abund_oct21 <- microbiome::transform(october2021,

transform = "compositional",

target = "OTU", shift = 0,

scale = 1)

input_data_oct21 <- as.data.frame(analysisdata_pruned_abund_oct21@otu_table) #extract ASV table from that new transformed dataset

input_data_oct21 <- as.data.frame(t(input_data_oct21)) #transpose to correct orientation

rownames(input_data_oct21) <- as.factor(oct2021_taxtable$ID)

meta_oct21 <- as.matrix(analysisdata_pruned_abund_oct21@sam_data) #extract metadata

meta_oct21 <- as.data.frame(meta_oct21) #make metadata a dataframe

fit_data_oct21 = Maaslin2(input_data = input_data_oct21, input_metadata = meta_oct21, output = "maaslin2_output_oct21", fixed_effects = c("Temperature"),plot_heatmap = TRUE) #run maaslin

fit_data_df_oct21 <- as.data.frame(fit_data_oct21$results) #pull out results only from maaslin output

fit_data_df_sig_oct21 <- subset(fit_data_df_oct21, qval <= 0.25) #pull out only significant results using q <= 0.25

maaslin2_oct21 <- ggplot(fit_data_df_sig_oct21, aes(x=coef, y=feature, color = value, shape = value)) +

theme_classic() +xlab("Coefficient") +

geom_errorbar(aes(xmin=coef-stderr,xmax=coef+stderr), width=.2,position=position_dodge(0.25)) +

geom_point(size=3, position=position_dodge(0.25),aes(fill=value, color=value))+

ylab("") + ggtitle("")+

xlab("") + xlab("Coefficient") + ylab("Feature") + scale_fill_manual(values=c('orange'))+ scale_color_manual(values=c('orange')) +

scale_shape_manual(values=seq(0,10))+

theme(axis.title.x=element_text(size=10)) +

theme(axis.title.y=element_text(size=10)) +

theme(axis.text.x=element_text(size=10)) +

theme(axis.text.y=element_text(size=8)) +theme(legend.text=element_text(size=10)) +

theme(legend.title=element_text(size=10))

maaslin2_oct21 #view plot

#january 2023/ 2023 (same)

january2023 <- subset_samples(maaslin_2023, Month_Year %in% c("Jan_2023"))

sample_data(january2023)

write.csv(january2023@tax_table, 'jan2023_ASVs.csv')

jan2023_taxtable <- read.csv('jan2023_ASVs.csv', header=TRUE)

ID <- (1:7639)

jan2023_taxtable$ID<- ID

View(jan2023_taxtable)

analysisdata_pruned_abund_jan23 <- microbiome::transform(january2023,

transform = "compositional",

target = "OTU", shift = 0,

scale = 1)

input_data_jan23 <- as.data.frame(analysisdata_pruned_abund_jan23@otu_table) #extract ASV table from that new transformed dataset

input_data_jan23 <- as.data.frame(t(input_data_jan23)) #transpose to correct orientation

rownames(input_data_jan23) <- as.factor(jan2023_taxtable$ID)

meta_jan23 <- as.matrix(analysisdata_pruned_abund_jan23@sam_data) #extract metadata

meta_jan23 <- as.data.frame(meta_jan23) #make metadata a dataframe

fit_data_jan23 = Maaslin2(input_data = input_data_jan23, input_metadata = meta_jan23, output = "maaslin2_output_jan23", fixed_effects = c("Temperature"),plot_heatmap = TRUE) #run maaslin

fit_data_df_jan23 <- as.data.frame(fit_data_jan23$results) #pull out results only from maaslin output

fit_data_df_sig_jan23 <- subset(fit_data_df_jan23, qval <= 0.25) #pull out only significant results using q <= 0.25

maaslin2_jan23 <- ggplot(fit_data_df_sig_jan23, aes(x=coef, y=feature, color = value, shape = value)) +

theme_classic() +xlab("Coefficient") +

geom_errorbar(aes(xmin=coef-stderr,xmax=coef+stderr), width=.2,position=position_dodge(0.25)) +

geom_point(size=3, position=position_dodge(0.25),aes(fill=value, color=value))+

ylab("") + ggtitle("")+

xlab("") + xlab("Coefficient") + ylab("Feature") + scale_fill_manual(values=c('orange'))+ scale_color_manual(values=c('orange')) +

scale_shape_manual(values=seq(0,10))+

theme(axis.title.x=element_text(size=10)) +

theme(axis.title.y=element_text(size=10)) +

theme(axis.text.x=element_text(size=10)) +

theme(axis.text.y=element_text(size=8)) +theme(legend.text=element_text(size=10)) +

theme(legend.title=element_text(size=10))

maaslin2_jan23

#Maaslin across years stats

#high

maaslin_high <- subset_samples(temp_subset_2, Temperature %in% c("high"))

sample_data(maaslin_high)

write.csv(maaslin_high@tax_table, 'maaslin_high.csv')

ma_high_taxtable <- read.csv('maaslin_high.csv', header=TRUE)

ID <- (1:7639)

ma_high_taxtable$ID<- ID

View(ma_high_taxtable)

analysisdata_pruned_abund_high <- microbiome::transform(maaslin_high,

transform = "compositional",

target = "OTU", shift = 0,

scale = 1)

input_data_high <- as.data.frame(analysisdata_pruned_abund_high@otu_table) #extract ASV table from that new transformed dataset

input_data_high <- as.data.frame(t(input_data_high)) #transpose to correct orientation

rownames(input_data_high) <- as.factor(ma_high_taxtable$ID)

meta_high <- as.matrix(analysisdata_pruned_abund_high@sam_data) #extract metadata

meta_high <- as.data.frame(meta_high) #make metadata a dataframe

fit_data_high = Maaslin2(input_data = input_data_high, input_metadata = meta_high, output = "maaslin2_output_high", fixed_effects = c("Year"), plot_heatmap = TRUE) #run maaslin

#low

maaslin_low <- subset_samples(temp_maaslin, Temperature %in% c("low"))

sample_data(maaslin_low)

write.csv(maaslin_low@tax_table, 'maaslin_low.csv')

ma_low_taxtable <- read.csv('maaslin_low.csv', header=TRUE)

ID <- (1:7639)

ma_low_taxtable$ID<- ID

View(ma_low_taxtable)

analysisdata_pruned_abund_low <- microbiome::transform(maaslin_low,

transform = "compositional",

target = "OTU", shift = 0,

scale = 1)

input_data_low <- as.data.frame(analysisdata_pruned_abund_low@otu_table) #extract ASV table from that new transformed dataset

input_data_low <- as.data.frame(t(input_data_low)) #transpose to correct orientation

rownames(input_data_low) <- as.factor(ma_low_taxtable$ID)

meta_low <- as.matrix(analysisdata_pruned_abund_low@sam_data) #extract metadata

meta_low <- as.data.frame(meta_low) #make metadata a dataframe

fit_data_low = Maaslin2(input_data = input_data_low, input_metadata = meta_low, output = "maaslin2_output_low", fixed_effects = c("Year"),plot_heatmap = TRUE) #run maaslin

#Maaslin across months stats

#high

maaslin_months_h

write.csv(maaslin_months_h@tax_table, 'maaslin_months_high.csv')

ma_high_months_taxtable <- read.csv('maaslin_months_high.csv', header=TRUE)

ID <- (1:7639)

ma_high_months_taxtable$ID<- ID

View(ma_high_months_taxtable)

analysisdata_pruned_abund_high_months <- microbiome::transform(maaslin_months_h,

transform = "compositional",

target = "OTU", shift = 0,

scale = 1)

input_data_high_months <- as.data.frame(analysisdata_pruned_abund_high_months@otu_table) #extract ASV table from that new transformed dataset

input_data_high_months <- as.data.frame(t(input_data_high_months)) #transpose to correct orientation

rownames(input_data_high_months) <- as.factor(ma_high_months_taxtable$ID)

meta_high_months <- as.matrix(analysisdata_pruned_abund_high_months@sam_data) #extract metadata

meta_high_months <- as.data.frame(meta_high_months) #make metadata a dataframe

meta_high_months$month_year2 <- as.factor(meta_high_months$month_year2)

meta_high_months$month_year2 <- relevel(meta_high_months$month_year2, ref = "Feb_2019")

fit_data_high_months = Maaslin2(input_data = input_data_high_months, input_metadata = meta_high_months, output = "maaslin2_output_high", fixed_effects = c("month_year2"), plot_heatmap = TRUE) #run maaslin

#Maaslin across months

#low

maaslin_low <- subset_samples(temp_maaslin, Temperature %in% c("low"))

maaslin_months_low <- subset_samples(maaslin_low, month_year2 %in% c("Feb_2019", "May_2019", "2020", "July_2021", "Oct_2021", "Jan_2023"))

maaslin_months_low

write.csv(maaslin_months_low@tax_table, 'maaslin_months_low.csv')

ma_low_months_taxtable <- read.csv('maaslin_months_low.csv', header=TRUE)

ID <- (1:7639)

ma_low_months_taxtable$ID<- ID

View(ma_low_months_taxtable)

analysisdata_pruned_abund_low_months <- microbiome::transform(maaslin_months_low,

transform = "compositional",

target = "OTU", shift = 0,

scale = 1)

input_data_low_months <- as.data.frame(analysisdata_pruned_abund_low_months@otu_table) #extract ASV table from that new transformed dataset

input_data_low_months <- as.data.frame(t(input_data_low_months)) #transpose to correct orientation

rownames(input_data_low_months) <- as.factor(ma_low_months_taxtable$ID)

meta_low_months <- as.matrix(analysisdata_pruned_abund_low_months@sam_data) #extract metadata

meta_low_months <- as.data.frame(meta_low_months) #make metadata a dataframe

meta_low_months$month_year2 <- as.factor(meta_low_months$month_year2)

meta_low_months$month_year2 <- relevel(meta_low_months$month_year2, ref = "Feb_2019")

fit_data_low_months = Maaslin2(input_data = input_data_low_months, input_metadata = meta_low_months, output = "maaslin2_output_low", fixed_effects = c("month_year2"), plot_heatmap = TRUE) #run maaslin

#maaslin mastergraph for all significant ASVs

masterfile <- read.csv("masterfile_update.csv")

sample_data(masterfile)

sample_data(masterfile)$value <- as.factor(sample_data(masterfile)$value)

maaslin2_mastergraph2 <- ggplot(masterfile, aes(x=coef, y=feature, color = as.factor(value), shape = as.factor(value))) +

theme_classic() +xlab("Coefficient") +

geom_errorbar(aes(xmin=coef-stderr,xmax=coef+stderr), width=.2,

position=position_dodge(0.25)) +

geom_point(size=7, position=position_dodge(0.25), shape= 16)+

ylab("") + ggtitle("")+

xlab("") + xlab("Coefficient") + ylab("") +

geom_vline(xintercept = 0, linetype = "dotted") +

scale_shape_manual(values=seq(0,10))+

theme(axis.title.x=element_text(size=28)) +

theme(axis.title.y=element_text(size=28)) +

theme(axis.text.x=element_text(size=28)) +

theme(axis.text.y=element_text(size=28)) +

theme(legend.text=element_text(size=28)) +

theme(legend.title=element_text(size=28))

maaslin2_mastergraph2 + labs(color = "Timepoint")

**Figure 6**

#prep for picrust2, https://github.com/picrust/picrust2/issues/136#issuecomment-743696114

#Change seq headers to asv number (ASV_1, ASV_2...)

asvs = as(otu_table(soil_relativeabund_pruned), "matrix") #read in soil RDS for relative abundance

asv_seqs <- colnames(asvs) #extract ASV namesasv

#Change seq headers to asv number (ASV_1, ASV_2...)

asv_headers <- vector(dim(asvs)[2], mode="character")

for (i in 1:dim(asvs)[2]) {

asv_headers[i] <- paste(">ASV", i, sep="_")

}

asvs_t <- t(asvs)

row.names(asvs_t) <- sub(">", "", asv_headers)

asv_df = as.data.frame(asvs_t)#made a transformed ASV table

write.table(asv_df, "Soil_ASVs_2023.tsv", quote=FALSE, sep='\t', col.names = NA)

#extract asv sequences only from asvs from soil.

#Create and write out a fasta of our final ASV seqs

asv_fasta <- c(rbind(asv_headers, asv_seqs))

write(asv_fasta, "Soil_ASVs.fa")

#picrust in terminal: When in your home folder, run the following

eval "$(/opt/anaconda3/bin/conda shell.bash hook)"

conda activate picrust2

picrust2_pipeline.py -s SoilASVs.fa -i Soil_ASVs_2023.tsv -o picrust2_out_pipeline2 -p 1

#Picrust analysis with maaslin

library(funrar)

library(Maaslin2)

pred_metagenome <- read.table('pred_metagenome_unstrat.tsv.gz', row.names = 1, header = 1)

pred_metagenome <- t(pred_metagenome)

pred_metagenome <- make_relative(pred_metagenome)

pred_metagenome <- as.data.frame(pred_metagenome) #formating and conversion to dataframe

pred_metagenome_maaslin <- subset(pred_metagenome, rownames(pred_metagenome)%in%rownames(Soil@sam_data))

meta <- as.matrix(soil_relativeabund_pruned@sam_data) # Soil = soil for relative abundance file from before, extracting meta data from this file.

meta <- as.data.frame(meta)

pred_metagenome_maaslin$Irrigation <- meta$Irrigation #add these to your picrust KO file so you can take these samples out

pred_metagenome_maaslin$Month_Year <- meta$Month_Year #add to your picrust KO file so you can make subsets

pred_metagenome_maaslin$Temperature <- meta$Temperature

pred_metagenome_maaslin$Burn <- meta$Burn

Subset_soil_noirrigation <- subset(pred_metagenome_maaslin, Irrigation %in% c("No")) #take out irrigated samples

Subset_soil_noirrigation_temp <- subset(Subset_soil_noirrigation, Temperature %in% c("high", "low")) #take out irrigated samples

Subset_soil_noirrigation_temp_burn <- subset(Subset_soil_noirrigation_temp, Burn %in% c("Burned", "Burnd")) #take out irrigated samples

#final files

write.table(Subset_soil_noirrigation_temp_burn, "KOs_2024.txt") #pathway file

write.table(Subset_soil_noirrigation_temp_burn, "pathways_2024.txt", row.names=TRUE, sep="\t") #KO file

library(ggpicrust2)

library(maaslin2)

meta_total <- read_delim("meta_picrust_years.txt", delim = "\t", escape_double = FALSE, trim_ws = TRUE)

pathways_total <- read_delim("pathways_2024_relative_transposed.txt", delim = "\t", col_names = TRUE, trim_ws = TRUE) #transposed on lilac but then had to transpose back in excel. See lilac code for subsetting of the metacyc. Made relative before subsetting.

S2019p <- pathways_total[ c(1:9) ]

Sfeb2019p <- pathways_total[ c(1:5) ]

Smay2019p <- pathways_total[ c(1,6:9) ]

S2020p<- pathways_total[ c(1, 10:12, 32:39) ]

S2021p<- pathways_total[ c(1,13:17, 40:44) ]

Sjuly2021p<- pathways_total[ c(1, 40:44) ]

Soct2021p<- pathways_total[ c(1, 13:17) ]

pathway_daa_2019 <- pathway_daa(abundance = S2019p %>% column_to_rownames("pathway"), metadata = meta_total, group = "Temperature", daa_method = "Maaslin2") #run for each subset

#combined all subsets in excel and then plotted all years/month_year sig pathways on a master graph (master_metacyc)

#plot

master_metacyc <- read_delim("maaslin_output_master_metacyc.txt", delim = "\t", col_names = TRUE, trim_ws = TRUE) #manually annotated these.

master_graph_metacyc <- ggplot(master_metacyc, aes(x=coef, y=Pathway, color = value, shape = General_Pathway)) +

+ theme_classic() +xlab("Coefficient") +

+ geom_errorbar(aes(xmin=coef-stderr,xmax=coef+stderr), width=.2,

+ position=position_dodge(0.25)) +

+ geom_point(size=3, position=position_dodge(0.25))+

+ ylab("") + ggtitle("")+

+ xlab("") + xlab("Coefficient") + ylab("") +

+ scale_shape_manual(values=seq(0,50))+

+ theme(axis.title.x=element_text(size=14)) +

+ theme(axis.title.y=element_text(size=10)) +

+ theme(axis.text.x=element_text(size=12)) +

+ theme(axis.text.y=element_text(size=12)) +

+ theme(legend.text=element_text(size=14)) +

+ theme(legend.title=element_text(size=14))

#high and low subsets for maaslin across years

highp <- pathways_total[ c(1,2,4,6, 8, 10, 11, 13:15, 18, 19, 22:25, 32, 33, 38, 41, 43) ]

lowp <- pathways_total[ c(1,3,5,7, 9, 12, 16, 17, 20, 21, 26:31, 34:37, 39, 40, 42,44) ]

meta_low <- subset(meta_total, Temperature %in% c("low"))

meta_low <- subset(meta_low, Irrigation %in% c("No")) #create low meta data file

lowp_pathways <- lowp[c(2:24)] #pull out only pathways, no pathways names

write.csv(lowp_pathways, "lowp_pathways.csv") #save as csvs, transpose pathways

write.csv(meta_low, "meta_low.csv")

df_meta_data_low = read.table(file = "meta_low.txt", header = TRUE, sep = "\t",

+ row.names = 1,

+ stringsAsFactors = FALSE)

df_input_low = read.table(file = "lowp_pathways_new.txt", header = TRUE, sep = "\t",

+ row.names = 1,

+ stringsAsFactors = FALSE) #input these files into R after editing them so sample_name is first column

df_meta_data_low$Year <- as.factor(df_meta_data_low$Year) #make Year a factor not a number

fit_data = Maaslin2(

+ input_data = df_input_low,

+ input_metadata = df_meta_data_low,

+ output = "./Maaslin2_results_Temperature", #whatever folder you've been working in.

+ fixed_effects = c("Year"), plot_heatmap = TRUE) #run maaslin2

#for high, 2 sig pathways in 2021 and 2023 relative to 2019, in low there were no sig pathways

#all KO subsets

KOs_total <- read_delim("KOs_2024.txt", delim = "\t", col_names = TRUE, trim_ws = TRUE)

S2019k <- KOs[ c(2:9) ]

Sfeb2019k <- KOs[ c(2:5) ]

Smay2019k <- KOs[ c(6:9) ]

S2020k<- KOs[ c(10:12, 32:39) ]

S2021k<- KOs[ c(13:17, 40:44) ]

Sjuly2021k<- KOs[ c(40:44) ]

Soct2021k<- KOs[ c(13:17) ]

highk <- KOs[ c(2,4,6, 8, 10, 11, 13:15, 18, 19, 22:25, 32, 33, 38, 41, 43) ]

lowk <- KOs[ c(3,5,7, 9, 12, 16, 17, 20, 21, 26:31, 34:37, 39, 40, 42,44) ]

S2023k <- KOs[ c(18:31) ] #filter only KOs, no KO names

S2023kf <- S2023k[rowSums(S2023k)>0,] #filter out KOs that sum to zero for all samples

S2023kft <- t(S2023kf) #transpose

write.csv(meta_total, "meta_total.csv") #write out meta data

meta_total = read.table(file = "meta_total.txt", header = TRUE, sep = "\t",

row.names = 1,

stringsAsFactors = FALSE) #read meta data back in after removing first row

#high v low

fit_data = Maaslin2(

input_data = S2023kft,

input_metadata = meta_2023,

output = "./Maaslin2_results_KOTemperature", #whatever folder you've been working in.

fixed_effects = c("Temperature"), plot_heatmap = TRUE) #run maaslin

#month year analysis, change to Year for just year.

fit_data = Maaslin2(

input_data = highkft,

input_metadata = meta_total,

output = "./Maaslin2_results_KOmonthyear", #whatever folder you've been working in.

fixed_effects = c("Month_Year2"), plot_heatmap = TRUE, reference="Feb_2019")

#month year

meta_total$Month_Year2 <- as.factor(meta_total$Month_Year2)

fit_data = Maaslin2(

input_data = highkft,

input_metadata = meta_total,

output = "./Maaslin2_results_KOmonthyear", fixed_effects = c("Month_Year"), reference = c("Feb_2019"), plot_heatmap = TRUE) #run maaslin

**#Tables 1 and 2, File S9**

library(psych)

#feb 2019

Sfeb2019_alphadiv <- read.csv("Sfeb2019alphadiv_corr.csv", header = TRUE)

Sfeb2019_pathway <- read.csv("Sfeb2019_pathway_corr_CURRENT.csv", header = TRUE)

Sfeb2019_pathwaysub <- Sfeb2019_pathway[c(2)] #select only numeric columns

Sfeb2019_corr <- corr.test(Sfeb2019_alphadiv, Sfeb2019_pathway[ c(2) ], method="spearman", ci=FALSE) #can integrate numerical selection with corr.test

S2019_padj <- as.data.frame(as.table(Sfeb2019_corr$p.adj))

p <- as.data.frame(as.table(Sfeb2019_corr$p))

r <- as.data.frame(as.table(Sfeb2019_corr$r))

Sfeb2019_corr_table <- cbind(r, p[,3], S2019_padj[,3]) #nothing sig for feb 2019

#tried this for high and low subsets but NaNs produced for all, too few samples. Same for ASVs

Sfeb2019_corr <- corr.test(Sfeb2019_ASVs_corr_total[ c(2)], Sfeb2019_pathway[ c(2) ], method="spearman", ci=FALSE)

#nothing sig when all samples included for ASVs.

#2020

S2020_corr <- corr.test(S2020_alphadiv[ c(2:3) ], S2020_pathway[ c(2:3) ], method="spearman", ci=FALSE) #high and low together

padj <- as.data.frame(as.table(S2020_corr$p.adj))

p <- as.data.frame(as.table(S2020_corr$p))

r <- as.data.frame(as.table(S2020_corr$r))

S2020_corr_table <- cbind(r, p[,3], padj[,3]) #sig for both pathways

#ASVs

S2020_ASVs_corrhigh<- read.csv("S2020_ASVs_corrhigh.csv", header = TRUE)

S2020_ASVs_corrlow<- read.csv("S2020_ASVs_corrlow.csv", header = TRUE)

S2020_corrhigh <- corr.test(S2020_ASVs_corrhigh[c(2:3)], S2020_pathway_corr_high[ c(2:3) ], method="spearman", ci=FALSE) #selected for numeric on pathway this way instead

padj <- as.data.frame(as.table(S2020_corrhigh$p.adj))

p <- as.data.frame(as.table(S2020_corrhigh$p))

r <- as.data.frame(as.table(S2020_corrhigh$r))

S2020_corrhigh_table <- cbind(r, p[,3], padj[,3])

View(S2020_corrhigh_table)

write.csv(S2020_corrhigh_table, "S2020_corrhigh_table.csv") #sig for multiple ASVs

#high and low separate

S2020_pathwaycorr_low<- read.csv("S2020pathway_corr_low.csv", header = TRUE)

S2020alphadiv_corr_low <- read.csv("S2020_alphadiv_corr_low.csv", header = TRUE)

S2020_corr_low <- corr.test(S2020alphadiv_corr_low[ c(2:3) ], S2020_pathwaycorr_low[ c(2:3) ], method="spearman", ci=FALSE)

padj <- as.data.frame(as.table(S2020_corr_low$p.adj))

p <- as.data.frame(as.table(S2020_corr_low$p))

r <- as.data.frame(as.table(S2020_corr_low$r))

S2020_corrlow_table <- cbind(r, p[,3], padj[,3])

View(S2020_corrlow_table) #chao1 and shannon positively correlated with pwy 6992 only in high soil.

#Oct 2021

Soct2021_corr <- corr.test(Soct2021_alphadiv[ c(2:3) ], Soct2021_pathway[ c(2:38) ], method="spearman", ci=FALSE)

padj <- as.data.frame(as.table(Soct2021_corr$p.adj))

p <- as.data.frame(as.table(Soct2021_corr$p))

r <- as.data.frame(as.table(Soct2021_corr$r))

Soct2021_corr_table <- cbind(r, p[,3], padj[,3]) #nothing sig for oct2021

Soct2021_pathway_corr_high<- read.csv("Soct2021_pathway_corr_high.csv", header = TRUE)

Soct2021alphadiv_corr_high <- read.csv("Soc2021_alphadiv_corr_high.csv", header = TRUE)

Soct2021_corr_high <- corr.test(Soct2021alphadiv_corr_high[ c(2:3) ], Soct2021_pathway_corr_high[ c(2:38) ], method="spearman", ci=FALSE)

padj <- as.data.frame(as.table(Soct2021_corr_high$p.adj))

p <- as.data.frame(as.table(Soct2021_corr_high$p))

r <- as.data.frame(as.table(Soct2021_corr_high$r))

Soct2021_corrhigh_table <- cbind(r, p[,3], padj[,3])

View(Soct2021_corrhigh_table) #a few with p adju = 0.

#for asvs, low has too few samples, all NaNs produced. For high, a few are sig.

Soct2021_corr <- corr.test(Soct2021_ASVs_corr_total[ c(2:20) ], Soct2021_pathway[ c(2:38) ], method="spearman", ci=FALSE)

#for ASVs including all, a few are sig.

#2023

S2023_corr <- corr.test(S2023_alphadiv[ c(2:3) ], S2023_pathway[ c(2:30) ], method="spearman", ci=FALSE)

padj <- as.data.frame(as.table(S2023_corr$p.adj))

p <- as.data.frame(as.table(S2023_corr$p))

r <- as.data.frame(as.table(S2023_corr$r))

S2023_corr_table <- cbind(r, p[,3], padj[,3]) #2 are below 0.1 but nothihg below 0.05

#nothing sig for 2023 high or low, ran fine but all adj p = 1 or NA

#nothing sig for either with ASvs

S2023_corr <- corr.test(S2023_ASVs_corr_total[ c(2:6) ], S2023_pathway[ c(2:30) ], method="spearman", ci=FALSE)

#nothing for ASVS including all samples
